# Excitatory somatostatin interneurons in the dentate gyrus drive a widespread seizure network in cortical dysplasia

**DOI:** 10.1038/s41392-023-01404-9

**Published:** 2023-05-17

**Authors:** Yang Zheng, Cenglin Xu, Jinyi Sun, Wenjie Ming, Sijie Dai, Yuying Shao, Xiaoyun Qiu, Menghan Li, Chunhong Shen, Jinghong Xu, Fan Fei, Jiajia Fang, Xuhong Jiang, Guoqing Zheng, Weiwei Hu, Yi Wang, Shuang Wang, Meiping Ding, Zhong Chen

**Affiliations:** 1 Department of Neurology, Zhejiang Provincial Hospital of Chinese Medicine, The First Affiliated Hospital of Zhejiang Chinese Medical University, Hangzhou, 310060 China; 2 Key Laboratory of Neuropharmacology and Translational Medicine of Zhejiang Province, School of Pharmaceutical Sciences, Zhejiang Chinese Medical University, Hangzhou, 310053 China; 3 Institute of Pharmacology and Toxicology, College of Pharmaceutical Sciences, Zhejiang University, Hangzhou, 310058 China; 4 Epilepsy Center, Department of Neurology, Second Affiliated Hospital Zhejiang University School of Medicine, Hangzhou, 310009 China; 5 Department of Neurology, Fourth Affiliated Hospital of Zhejiang University School of Medicine, Yiwu, 322000 China

**Keywords:** Neurodevelopmental disorders, Diseases of the nervous system

## Abstract

Seizures due to cortical dysplasia are notorious for their poor prognosis even with medications and surgery, likely due to the widespread seizure network. Previous studies have primarily focused on the disruption of dysplastic lesions, rather than remote regions such as the hippocampus. Here, we first quantified the epileptogenicity of the hippocampus in patients with late-stage cortical dysplasia. We further investigated the cellular substrates leading to the epileptic hippocampus, using multiscale tools including calcium imaging, optogenetics, immunohistochemistry and electrophysiology. For the first time, we revealed the role of hippocampal somatostatin-positive interneurons in cortical dysplasia-related seizures. Somatostatin-positive were recruited during cortical dysplasia-related seizures. Interestingly, optogenetic studies suggested that somatostatin-positive interneurons paradoxically facilitated seizure generalization. By contrast, parvalbumin-positive interneurons retained an inhibitory role as in controls. Electrophysiological recordings and immunohistochemical studies revealed glutamate-mediated excitatory transmission from somatostatin-positive interneurons in the dentate gyrus. Taken together, our study reveals a novel role of excitatory somatostatin-positive neurons in the seizure network and brings new insights into the cellular basis of cortical dysplasia.

## Introduction

Seizures in cortical dysplasia (CD) tend to generalize via a large-scale seizure network, despite their localized anomalies.^1^ This is particularly a concern in late-stage CD, defined as cortical malformations originating from the post-migration synaptogenesis stage, which typically include focal cortical dysplasia (FCD) type I, polymicrogyria and gray matter heterotopia.^2^ Late-stage CD represents one of the most common causes of pharmacoresistant epilepsy.^3,4^ Notably, post-surgical relapses often arise from sources distant to the original focus, presumably due to disruption of brain connectivities.^5–7^ The notion of widespread seizure network is further supported by its unclear lesion margins and frequent cognitive comorbidities.^3,5,6^ However, it remains unclear what drives the widespread seizure network in late-stage CD.

While most investigations have focused on primary lesions in the neocortex of CD, the functional role of the remote area is less characterized. Abnormalities of the remote hippocampus, however, were frequently observed. Incomplete inversion/ hypoplasia of the hippocampus, as well as hippocampal hyperconnections, have been reported in patients with late-stage CD.^7–11^ These studies left open the question how the hippocampus, as a remote brain region, contributes to the extensive seizure network in late-stage CD.

More specific defects have been reported in GABAergic interneurons, though studies are mostly limited to histopathological changes within resected lesions.^12–14^ Impairment in the GABAergic system may result from normal GABA receptors participating in abnormal circuitry,^15^ loss of a subpopulation of GABAergic interneurons,^16^ or changes in intrinsic cellular properties.^17^ Among the various subtypes, a reduction of parvalbumin (PV) + ^12,13,18^ and somatostatin (SOM) + ^12^ interneurons was reported in surgical specimens of FCD patients, though their functions remains largely unexplored. Advances in multi-level approaches allow visualization and manipulation of remote interneurons in the seizure network.^19^ A recent study reported the crucial role of remote SOM + interneurons in CD-related seizures,^19^ further motivating us to explore the interneuron subtypes outside the dysplastic regions in cortical malformation.

In this study, we tackled the question of a widespread seizure network in late-stage CD. We hypothesized that the hippocampal interneurons serve as a crucial gate in the network. To test this hypothesis, we first provided clinical evidence that epileptogenicity of the hippocampus was highly associated with the widespread seizure network in late-stage CD. Next, we revealed an unexpected excitatory effect of SOM + interneurons on seizure generalization in the late-stage CD rat model,^20,21^ mainly attributed to neurotransmitter phenotype switching. Taken together, our results provide evidence and propose a novel mechanism reconciling the excitatory hippocampal interneurons and the widespread seizure network in late-stage CD.

## Results

### Involvement of hippocampus in the seizure network of late-stage CD

**Fig. 1:**
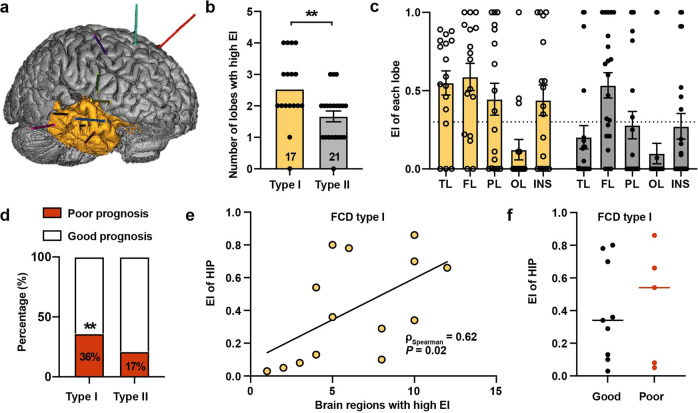
Epileptogenicity of the hippocampus was associated with the extension of the seizure network in focal cortical dysplasia (FCD) type I. **a** Representative scheme of presurgical stereo-electroencephalography recording. Intracerebral electrodes, labeled in different colors, were used to measure epileptogenicity in different brain regions in the sample patient. The yellow shaded area represented the resected region. **b** Patients with FCD type I had a more widespread seizure network than those with FCD type II, as denoted by more brain lobes with a high epileptogenicity index (EI > 0.3). Mann–Whitney test was used. **c** In FCD type I, there were more lobes with an EI > 0.3 (dashed line), in comparison to FCD type II where only the frontal lobe had a high EI. **d** Patients with FCD type I were more likely to have a poor postsurgical prognosis (Engel class II-IV) than those with FCD type II. Chi-square test was used. **e** Among patients with FCD type I, a significant relationship was found between the EI value of the hippocampus and the number of brain regions with a high EI (ρ_Spearman_ = 0.62 [95% confidence interval 0.12–0.87], *P* = 0.02). Correlation analysis was performed. **f** Among patients with FCD type I, the EI value of the hippocampus was numerically higher in those with a poor prognosis (Engel class II-IV) than those with a good one (Engel class I). Mann–Whitney test was used. *P < 0.05, **P < 0.05

We included patients with FCD type I as a representative of late-stage CD, and type II representing early-stage CD. Fifty patients who underwent stereo-electroencephalography (SEEG) recordings were further considered for quantitative analysis (Fig. 1a). Twelve patients were excluded due to the impossibility of computing the epileptogenicity index (EI) (absence of fast discharges [*n* = 7] or noisy signals [*n* = 5]). Finally, 38 patients (17 with type I and 21 with type II) with a total of habitual seizures (2 habitual seizures per patient) were included for SEEG analysis (Supplementary Table 1).

Visual analysis revealed that the hippocampus tended to participate in seizures of FCD type I, compared to those with type II (29.4% [5/17] vs. 9.5% [2/21] in the first 15 seconds). With quantitative analysis, 47.1% (8/17) showed high values of hippocampal epileptogenicity (EI > 0.3) in patients with FCD type I, compared to only 19.0% in those with type II (4/21). As far as localization was concerned, a high level of hippocampal epileptogenicity was mainly found in patients with a temporal lobe onset (62.5% [5/8] in FCD type I and 60% [3/5] in FCD type II). Interestingly, even for those with an extratemporal onset, 33.3% [3/9] of patients with FCD type I had high hippocampal epileptogenicity, compared to only 6.3% [1/16] of patients with FCD type II.

We further investigated the association between the hippocampus and the extension of seizure network. The prognosis was analyzed in patients with at least a 1-year follow-up (14 with type I and 7 with type II). Firstly, we found that FCD type I had a much more widespread seizure network than type II (Number of brain regions with EI > 0.3, *P* = 0.009, Fig. 1b, c). Patients with FCD type I were more likely to have post-surgical relapses (Engel class II-IV) than those with FCD type II (*P* = 0.002, Fig. 1d). Next, we asked whether the hippocampus contributed to the widespread seizure network in FCD type I. A significant correlation was found between the extension of the seizure network and hippocampal epileptogenicity among patients with FCD type I (*P* = 0.02, Fig. 1e). For FCD type I, the average epileptogenicity of the hippocampus was numerically higher in those with a poor outcome (Engel class II-IV) than those with a good one (Engel class I) (*P* > 0.05, Fig. 1f). By contrast, for FCD type II, there was no correlation between hippocampal epileptogenicity and the extension of seizure network (*P* > 0.05) or surgical outcome (*P* > 0.05).

Taken together, we showed a correlation between hippocampal epileptogenicity and the widespread seizure network in FCD type I. Next, the underlying mechanism of hippocampal epileptogenicity was further investigated in the rat model of late-stage CD.

### Increased hippocampal excitability in CD rats

**Fig. 2:**
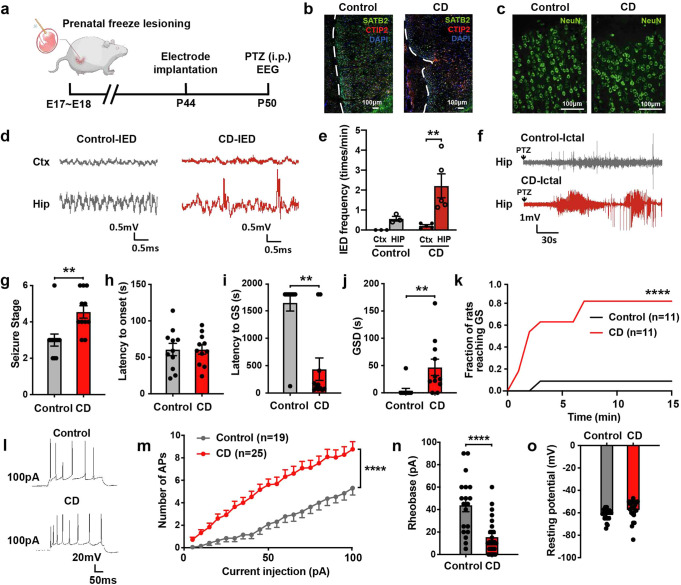
The prenatal freeze lesioned rat model recapitulated key features of patients with late-stage cortical dysplasia (CD). **a** Schematic of prenatal freeze lesioning on the uterus at the late embryonic age (embryonic age 17–18, E17–18). All experiments, including behavioral tests, electroencephalography (EEG) recording and immunofluorescence analysis were performed on postnatal day 50 (P50). **b** Immunostaining with SATB2 (green), CTIP2 (red), and DAPI (blue) antibodies demonstrated dyslamination in the cortex of prenatal freeze-lesioned rats. **c** Higher magnification views showing the abnormal polarity of the NeuN + (green) cells. **d** Representative EEG traces of the interictal epileptiform discharges in the hippocampus and cortex in control (gray) and CD rats (red). **e** Frequency of interictal epileptiform discharges (number of interictal epileptiform discharges / minutes) was compared between the cortex and hippocampus in control and CD rats. Mann–Whitney test was used. **f** Representative ictal intracranial EEG traces after intraperitoneal injection of pentylenetetrazol (PTZ) in the hippocampus of control (gray) and CD (red) rats. **g-k** Seizure severity in control (gray) and CD rats (red), as denoted by seizure stage (**g**), latency to onset (**h**), latency to generalized seizures (GS) (**i**), GS duration (GSD) (**j**) and seizure progression (**k**) after PTZ delivery. Mann–Whitney test was used for (**g**–**j)**. Two-way ANOVA was used for (**k**). **l** Representative traces of action potentials recorded by whole-cell patch clamp with an injected current of 100pA (control [upper], CD rat [lower]). **m** More spike numbers in granule cells of CD rats (red) were evoked by trains of stepped depolarized currents than controls (gray). Two-way ANOVA was used. **n, o** Granule cells in CD rats had a lower rheobase than those in control rats. The resting membrane potential of granule cells was comparable between the control and CD rats. *P < 0.05, **P < 0.01, ****P < 0.0001. Data are presented as mean ± s.e.m. and error bars represent s.e.m

To investigate the role of hippocampus in late-stage CD, we established the rat model with prenatal freeze-lesioning at the late embryonic stage, which recapitulated key features in late-stage CD patients (Fig. 2a and Supplementary Fig. S1a, b).^20,21^ Histopathologic changes included microgyria and cortical dyslamination (Fig. 2b, c and Supplementary Fig. S1c). Electroencephalography (EEG) recordings showed interictal rhythmic spikes, polyspikes and paroxysmal fast activities (Fig. 2d and Supplementary Fig. S1d) which resembled the interictal discharges in patients.^22^ There was a higher frequency of spontaneous interictal discharges in the hippocampus compared to the recorded cortex (Fig. 2e), indicating a high hippocampal excitability.

To evaluate seizure susceptibility in CD rats, we induced seizures with a subthreshold dose of pentylenetetrazol (PTZ). The low-dose PTZ caused only focal seizures in most controls (10 out of 11). Remarkably, it resulted in a higher seizure severity in most CD rats, with 9 out of 11 CD rats showing generalized seizures (GS) (Fig. 2f). The tendency of seizure genralization suggested a more widespread seizure network in our CD model.^23^ Seizures in CD rats also exhibited a shorter latency to GS and a prolonged GS duration (Fig. 2g–k). Whole-cell recordings in brain slices showed increased excitability of hippocampal dentate gyrus (DG) granule cells in CD rats (Fig. 2l–n). There were no significant differences in intrinsic membrane properties of granule cells between the groups, including the resting membrane potentials, action potential (AP) threshold, amplitude and half-width (Fig. 2o, Supplementary Fig. S2). In contrast, no significant changes in excitability were observed in hippocampal pyramidal cells of CA1 and subiculum (Supplementary Fig. S3), both of which were previously reported to be involved in seizures.^24,25^ Taken together, the prenatal freeze-lesioned rats recapitulated the key anatomical-clinical-electrophysiological features of late-stage CD. We also showed selective increase in excitability of the hippocampal DG in CD rats.

### Selective impairment of SOM + interneurons in the dentate gyrus of CD rats

**Fig. 3:**
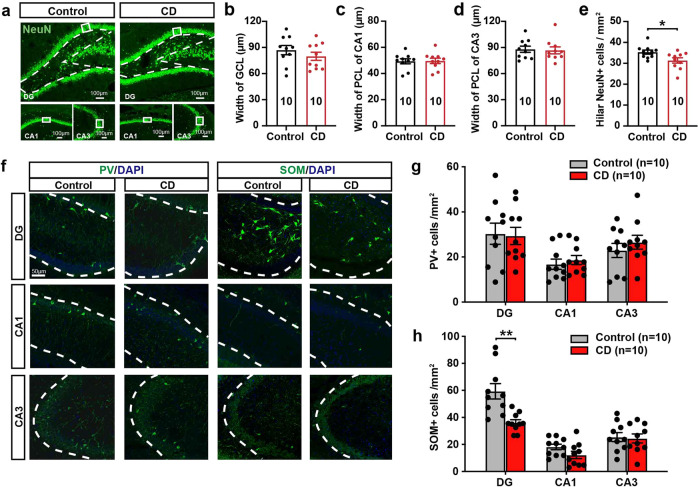
Selective loss of somatostatin (SOM)-positive interneurons in the dentate gyrus (DG) of cortical dysplasia (CD) rats. **a** Mature neurons within the hippocampus (DG, upper; CA1, lower left; CA3, lower right) of control and CD rats were shown via NeuN labeling (green). The width of granule cell layer (GCL) (**b**), pyramidal cell layer (PCL) of CA1 (**c**) and CA3 (**d**) was all comparable between control and CD rats. Areas boxed in white solid lines in (**a**) were used for data analysis. Mann–Whitney test was used. **e** In the hilus (regions in white dashed white lines in [**a**]), there was a significant reduction of NeuN+ cells in CD rats. Mann–Whitney test was used. **f** Representative fluorescence microscopy images of parvalbumin (PV) + and SOM + interneurons within the DG (hilus and granule cell layer), CA1 (stratum pyramidale [SP] and stratum radiatum), and CA3 (SP and stratum lucidum) of the hippocampus. **g** Quantification of the density of PV + and SOM + interneurons in the DG, CA1 and CA3 of the hippocampus. Multiple Mann–Whitney test with Holm-Sidak corrections was used. *P < 0.05, **P < 0.01. Data are presented as mean ± s.e.m. and error bars represent s.e.m

To investigate the histopathologic substrates of hippocampal epileptogenicity in CD rats, we analyzed the density and distribution of different cell types across 3 main subregions, i.e. DG, CA1 and CA3 (Fig. 3a). The overall NeuN+ cells were first investigated. Width of the granule cell layer (GCL) in the DG and pyramidal cell layer (PCL) in CA1 and CA3, predominantly composed of glutamatergic excitatory cells, remained unchanged (Fig. 3b–d). We found a significant reduction of NeuN+ cell density in the hilus, which includes a mix of glutamatergic cells and GABAergic interneurons (Fig. 3e). Further, we used calmodulin-dependent protein kinase II (CaMKII) staining to label the glutamatergic cells in the hippocampus. The width of CaMKII+ GCL of DG and PCL in CA1 and CA3, as well as the density of hilar CaMKII+ cells, were comparable between the groups (Supplementary Fig. S4). For interneurons, we used SOM and PV staining to label the 2 major subtypes in the hippocampus. The density of PV + interneurons remained unchanged in CD rats (Fig. 3f, g). Unexpectedly, we found a dramatic reduction of SOM + interneurons in the DG of CD rats, mostly within the hilus (Fig. 3f, h). To investigate the cause of the reduced density, we co-stained SOM with terminal deoxynucleotidyl transferase-dUTP nick end labeling (TUNEL) at different developmental stages of CD rats. There was a reduction in SOM density in CD rats from as early as P5, and markers of apoptosis were scarcely detected from P5 to P50 (Supplementary Fig. S5). These results might suggest an early developmental deficit in SOM + interneurons. Taken together, our morphological analysis revealed an early and selective loss of SOM + interneurons in the hippocampus of CD rats.

### Dentate gyrus SOM + interneurons facilitate the generalization of CD-related seizures

**Fig. 4:**
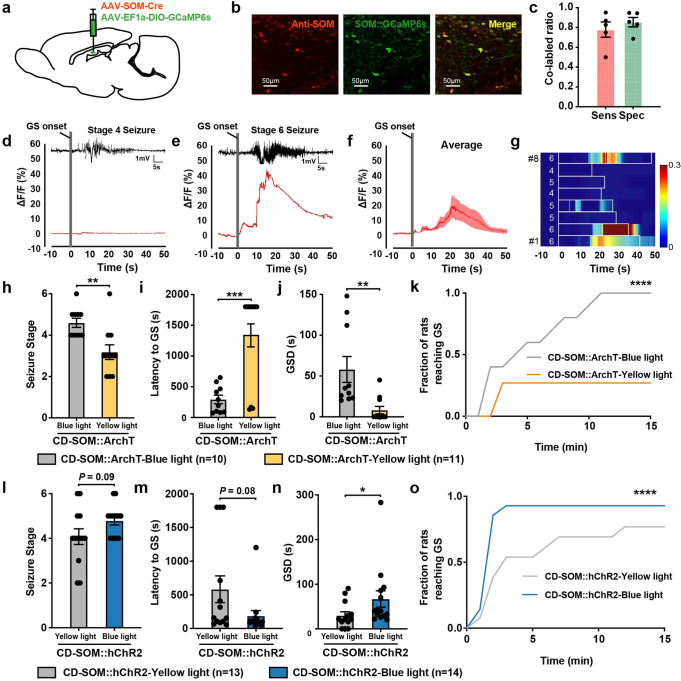
The hyperactive somatostatin (SOM) + interneurons facilitated seizure generalization in cortical dysplasia (CD) rats. **a** Scheme of experiment for viral cocktail (AAV-SOM-Cre and AAV-EF1a-DIO-GCaMP6s) injection into the dentate gyrus (DG) for calcium imaging experiments. **b** Representative images of DG showing the overlap (yellow) of SOM::GCaMP6s (green) and SOM + neurons (red). **c** The viral cocktail of SOM::GCaMP6s showed a sensitivity of 77.94 ± 7.7%, and a specificity of 85.37 ± 4.78%. Sensitivity was calculated as the number of merged neurons (yellow) over neurons labeled with anti-SOM antibodies (red). Specificity was calculated as the number of merged neurons (yellow) over neurons expressing the SOM::GCaMP6s viral cocktail (green). Representative EEG and corresponding peri-event plots of calcium signals of DG SOM + interneurons during stage 4 (**d**) and stage 6 pentylenetetrazol (PTZ)-induced generalized seizures (GS) (**e**). **f** The averaged peri-plots of calcium signals of DG SOM + interneurons aligned to the initiation of PTZ-induced GS in CD rats, revealing an increased activity of SOM + interneurons. A total of 8 GS from 5 CD rats were included. The red curve indicated the mean, and shaded areas indicated s.e.m. **g** Heatmap of calcium signals aligned to the initiation of PTZ-induced GS, showing a stage-dependent increase of SOM + interneuron activities. Each row represented the typical calcium signal of a single GS (boxed in white). The corresponding seizure stage was labeled in white numbers in the front. Color scale indicated *ΔF/F* and warmer colors indicated a higher fluorescence signal. Effects of optogenetic inhibition of DG SOM + neurons on the seizure stage (**h**), latency to GS onset (**i**), GS duration (GSD) (**j**), and seizure progression (**k**) after PTZ delivery. Mann–Whitney test was used for (**h**–**j**); Two-way ANOVA was used for (**k**). Effects of optogenetic activation of DG SOM + neurons on the seizure stage (**l**), latency to GS onset (**m**), GSD (**n**), and seizure progression (**o)** after PTZ delivery. Mann–Whitney test was used for (**l**–**n**); Two-way ANOVA was used for (**o**). *P < 0.05, **P < 0.01, ****P < 0.0001. Data are presented as mean ± s.e.m. and error bars represent s.e.m

As the recording of calcium signals is widely used for the monitoring of neuronal functional activity in vivo, we sought to evaluate the real-time activity of hippocampal interneurons during PTZ-induced seizures. We first transfected GCaMP6s in DG SOM + interneurons of CD rats (SOM::GCaMP6s rats, Fig. 4a–c, Supplementary Fig. S6a). After aligning EEG and photometry recordings after the GS onset, the calcium signals of SOM + interneurons increased with GS initiation, reached a peak during the mid of GS, and gradually faded back to the baseline after GS cessation. The increase, moreover, was seizure stage-dependent, with the greatest changes occurring in stage 6 (Fig. 4d, e). The dynamic calcium signals indicated that DG SOM + interneurons were hyperactive in CD-related GS.

To further address the role of DG SOM + interneurons in seizure generalization, we used ArchT to selectively inactivate SOM + interneurons in CD rats (SOM::ArchT rats, Supplementary Fig. S7a–c). Unexpectedly, inhibition of SOM + interneurons significantly abolished seizure generalization, as shown by fewer rats exhibiting GS, a shorter GS duration, and a rightward shift of the cumulative probability distribution for GS versus time after PTZ injection (Fig. 4h–k). This was in contrast to a slight exacerbation of seizures when inhibiting SOM + interneurons in control rats (Supplementary Fig. S8). Next, we used hChR2 to selectively activate SOM + interneurons during seizures (SOM::hChR2 rats, Supplementary Fig. S7d–f). Activation of SOM + cells in DG significantly accelerated seizure generalization in CD rats, causing prolonged GS duration and a leftward shift of the cumulative probability distribution curve (Fig. 4l–o). The initial spectral power, however, did not change upon SOM activation in CD rats (Supplementary Fig. S9), indicating a minor role of SOM + interneurons in seizure onset. Together, our results suggested that DG SOM + interneurons were increasingly recruited during GS and facilitated seizure generalization in late-stage CD.

### Dentate gyrus PV + interneurons inhibit CD-related seizures

**Fig. 5:**
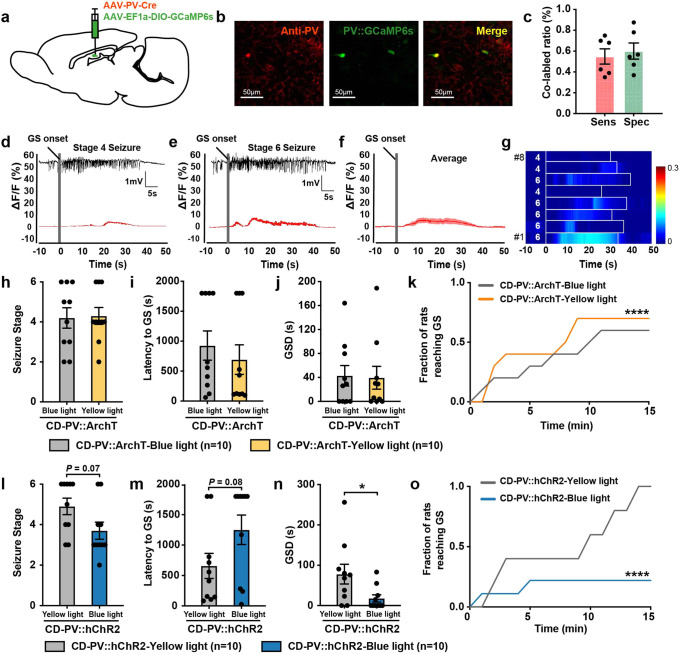
Parvalbumin (PV) + interneurons attenuated seizures in cortical dysplasia (CD) rats. **a** Scheme of experiment for viral cocktail injection (AAV-PV-Cre and AAV-EF1a-DIO-GCaMP6s) into the dentate gyrus (DG) for calcium imaging experiments. **b** Representative images of DG showing the overlap (yellow) of PV::GCaMP6s (green) and PV + neurons (red). **c** The viral cocktail of PV::GCaMP6s showed a sensitivity of 54.90 ± 7.32%, and a specificity of 60.09 ± 7.80%. Sensitivty was calculated as the number of merged neurons (yellow) over neurons labeled with anti-PV antibodies (red). Specificity was calculated as the number of merged neurons (yellow) over neurons expressing the PV::GCaMP6s viral cocktail (green). Representative EEG and corresponding peri-event plots of calcium signals of DG PV + interneurons during stage 4 (**d**) and stage 6 pentylenetetrazol (PTZ)-induced generalized seizures (GS) (**e**). **f** The averaged peri-plots of calcium signals of DG PV + interneurons aligned to the initiation of PTZ-induced GS in CD rats, revealing only a minimally increased activity of PV + interneurons. A total of 8 GS from 6 CD rats were included. The red curve indicated the mean and shaded areas indicated s.e.m. **g** Heatmap of calcium signals aligned to the initiation of PTZ-induced GS. Each row represented the typical calcium signal of a single GS (boxed in white). The corresponding seizure stage was labeled in white numbers. Color scale indicated △F/F and warmer colors indicated a higher fluorescence signal. Effects of optogenetic inhibition of DG PV + neurons on the seizure stage (**h**), latency to GS onset (**i**), GS duration (GSD) (**j**), and seizure progression (**k**) after PTZ delivery. Mann–Whitney test was used for (**h**–**j**). Two-way ANOVA was used for (**k**). Effects of optogenetic activation of DG PV + neurons on the seizure stage (**l**), latency to GS onset (**m**), GSD (**n**), and seizure progression (**o**) after PTZ delivery. Mann–Whitney test was used for (**l**–**n**); Two-way ANOVA was used for (**o**). *P < 0.05, ****P < 0.0001. Data are presented as mean ± s.e.m. and error bars represent s.e.m

Next, we investigated the calcium dynamics of DG PV + interneurons during CD-related GS by transfecting GCAMP6s to DG PV + interneurons in CD rats (PV::GCaMP6s rats, Fig. 5a–c, Supplementary Fig. S6b). Distinct from SOM + interneurons, the calcium signals in PV + interneurons had a weak response during CD-related GS. PV + interneurons remained hypoactive regardless of seizure stages (Fig. 5d–g). Further, we selectively modulated DG PV + interneurons during CD-related seizures with optogenetics (PV::ArchT rats or PV::hChR2 rats, Supplementary Fig. S10). PV inhibition worsened seizure progression in CD rats (Fig. 5h–k). PV activation led to a reduction of GS duration of CD-related seizures and slowed seizure progression (Fig. 5i–o). The overall effect of PV manipulations, however, was less evident when compared to that of SOM manipulations in CD rats. In addition, the inhibitory effect of PV + interneurons was also found in control rats, where PV activation slowed seizure progression as well (Supplementary Fig. S11). Taken together, these results indicated that PV + interneurons retained their inhibitory role in CD-related seizures.

### Excitatory SOM + interneurons in the dentate gyrus co-release GABA and glutamate in CD rats

**Fig. 6:**
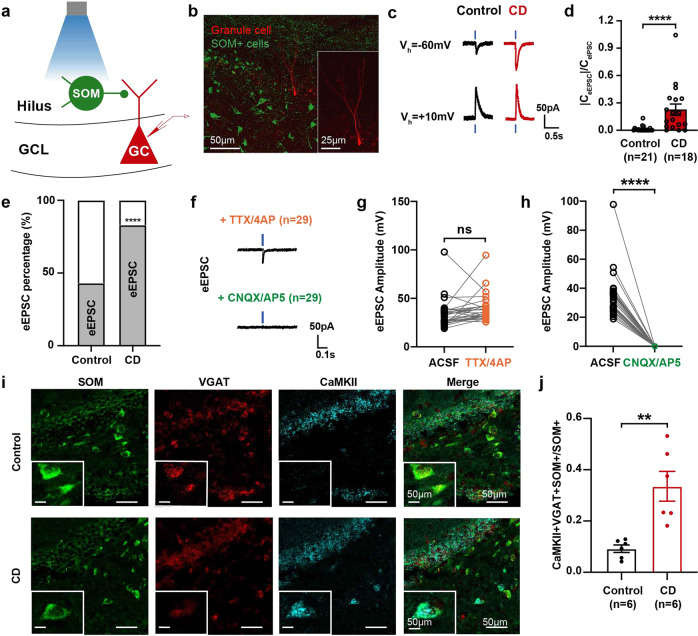
Increased glutamate co-release from somatostatin (SOM) + interneurons in cortical dysplasia (CD) rats. **a** Diagram of experimental protocol. Whole-cell recording of granule cells (GCs, red) upon optogenetic activation of SOM + neurons transfected with hChR2-EYFP (green). **b** Representative images of the recorded GC (filled with biocytin) in the DG. The optogenetically activated hChR2+ SOM-expressing neurons were labeled in green. **c** Representative traces of light-evoked excitatory postsynaptic currents (EPSCs) and inhibitory postsynaptic currents (IPSCs) in the GCs of control and CD rats. **d** Quantification of eEPSC/eIPSC charge transfer ratios in the granule cells of control and CD rats. Mann–Whitney test was used**. e** The response rate of light-evoked EPSCs in control and CD rats. Chi-square test was used. **f** Representative traces of eEPSCs in control and CD rats under tetrodotoxin (TTX) / 4-aminopyridine (4-AP) treatment. Further application with 6-cyano-7-nitroquinoxaline-2,3-dione (CNQX) and (2 R)-amino-5-phosphonovaleric acid (D-AP5) was able to block the eEPSCs completely in control and CD rats respectively. **g** Quantification of eEPSC amplitude pre- and post-TTX / 4AP application in control and CD rats. Paired *t* test was used. **h** Quantification of eEPSC amplitude pre- and post-CNQX/AP5 application in control and CD rats. Paired *t* test was used. **i** Representative images showing the co-staining of SOM (green), VGAT (red) and CaMKII (cyan) in control and CD rats. **j** Quantification of the proportion of DG SOM + interneurons co-expressing VGAT and CaMKII. Mann–Whitney test was used. *P < 0.05, **P < 0.01, ****P < 0.0001. Data are presented as mean ± s.e.m. and error bars represent s.e.m

We next examined the projections from hippocampal SOM + to granule cells with electrophysiological and anatomical approaches. Upon blue light stimulation, SOM + interneurons expressing hChR2 were optically activated. Simultaneously, evoked postsynaptic currents were recorded in nearby granule cells, and depolarizing and hyperpolarizing currents were distinguished with different holding potentials (Fig. 6a–d). When held at +10 mV, hyperpolarizing inhibitory postsynaptic currents (IPSCs) could be evoked in both groups. The granule cells were next held at −60 mV to delineate the light-evoked depolarizing excitatory postsynaptic currents (eEPSCs).^26^ In contrast to control rats showing only rare and minimal eEPSC responses, the response rate of eEPSCs and the eEPSC/eIPSC charge transfer ratio were significantly increased in CD rats (Fig. 6c–e). The postsynaptic currents were recorded in the presence of tetrodotoxin (TTX)/ 4-aminopyridine (4-AP), indicating a monosynaptic transmission (Fig. 6f, g).^27^ Further application with AMPA receptor antagonist 6-cyano-7-nitroquinoxaline-2,3-dione (CNQX, 20 μM) and NMDA receptor antagonist (2 R)-amino-5-phosphonovaleric acid (D-AP5, 50 μM) totally abolished the eEPSCs in CD rats (Fig. 6f, h). The findings suggested glutamate-mediated excitatory transmission from SOM + interneurons in CD rats. Apart from presynaptic changes, another possibility that underlies excitatory interneurons is a positive shift in the equilibrium potential for GABA_A_ receptors (E_GABA_) in the postsynaptic granule cells, which may lead to depolarizing GABA-mediated currents.^28^ Accordingly, we performed gramicidin-perforated patch recordings in the granule cells of the hippocampus in vitro, which showed an unchanged E_GABA_ in CD rats (Supplementary Fig. S12).

To examine anatomically whether GABA and glutamate can be released from individual neurons, we used a combination of fluorescence in situ and histochemical studies. In CD rats, around 33% of SOM + interneurons co-expressed CaMKII (markers for glutamatergic neurons) and VGAT (markers for GABAergic neurons). In control rats, CaMKII and VGAT displayed only minimal overlap in SOM + interneurons (Fig. 6i, j). Altogether, the results suggested an increased probability of glutamate release from SOM + interneurons in CD rats, resulting in a direct excitatory effect on downstream granual cells.

## Discussion

A major challenge in CD is its poor prognosis due to an uncharacterized widespread seizure network. Herein, we harnessed clinical and experimental studies to probe the cellular changes leading to the widespread seizure network in late-stage CD. Our data suggest that the hippocampus, though located remote from the dysplastic lesion, is a key determinant of the widespread seizure network. An increased glutamate release from SOM + interneurons constitutes the cellular substrate within the hyperexcitable hippocampus. As opposed to the conventional view emphasizing the role of internal dysregulation within the dysplasia lesion, our data point to the excitatory SOM + interneurons within the hippocampal DG as the major driving force of the widespread seizure network.

Hippocampal abnormalities have long been observed in patients with late-stage CD, though their significance remains debated.^8,9^ Our study was based on the observation that the extension of seizure network in late-stage CD was positively associated with epileptogenicity of the hippocampus. The finding was further echoed by previous studies revealing a functional hyperconnectivity of the hippocampus in patients with late-stage CD, which explained its tendency of recruitment in seizures.^10,29^ We chose the prenatal freeze lesioning model since it matched the key pathogenesis underlying late-stage CD, considering the form of injuries (physical/chemical) and timing at the late embryonic stage.^21^ The rat model exhibited spontaneous interictal discharges and higher excitability in the DG. Bidirectional modulation of the DG controled seizure generalization, reminiscent of large-scale network spread.^1,23^ The finding, however, may not be generalized to early-stage malformations such as FCD type II, where the seizure network is often localized and hippocampal abnormalities showed little relevance with the network extension.^9^ Altogether, the multiple lines of evidence suggest that hippocampal abnormalities might not be an entirely benign finding in late-stage malformations.

Why does the hippocampus, despite its anatomical location outside the dysplastic lesion, display increased excitability? Late-stage malformations is characterized by diffuse cortical thinning on structural MRI and rearrangement of whole-brain connectivity on functional imaging.^7,30^ As a richly connected structure, the hippocampus is susceptible to such widespread neurodevelopmental damage, which has been reported in periventricular nodular heterotopia, polymicrogyria and genetic generalized epilepsy syndromes.^10,31^ Therefore, we speculate that migrational or postmigrational abnormalities predisposing to the large-scale seizure network may overlap with determinants of hippocampal maldevelopment. Thus, an improved understanding of mechanisms underlying hippocampal excitability may enable discovery of therapeutic targets for widespread damage in late-stage malformations.^18,32^

Interneurons have been increasingly recognized to play major roles in the seizure network of CD.^33,34^ The exact nature of the disrupted inhibitory activity is not fully understood. We focus on the PV + and SOM + subtypes of interneurons, based on recent findings that they change in number in human surgical samples.^12^ In the CD model, SOM + interneurons were lost in the DG hilus, similar to previous observations in experimental and human temporal lobe epilepsy.^35,36^ The early decrease may suggest developmental defects of SOM + interneurons in CD, including impaired migration, differentiation or reduced proliferation.^37^ The remaining SOM + interneurons were hyperactive, and changes in SOM activity correlated with seizure severity. Optogenetic manipulations of SOM + interneurons had a greater impact on CD-related seizures than PV + interneurons. The findings strongly suggested the recruitment of SOM + interneurons in the seizure network in CD rats. Interestingly, contrary to the conventional view that interneurons take an inhibitory role, our current work revealed that SOM + interneurons in the DG, rather than PV + interneurons, facilitated seizure generalization, which resonated with the clinical findings of a widespread seizure network. However, the current study cannot totally exclude the role of other cell types in CD-related seizures, and future studies with targeted manipulations are still needed. Overall, our study identify an unusal form of interneuron deficits in the hippocampus controlling seizure generalization in late-stage cortical malformations.

How do the SOM + interneurons undergo the excitatory switch? We uncovered that a subset of SOM + interneurons in the DG might undergo neurotransmitter phenotype switching, a previously unreported phenomenon in cortical malformations. Despite the traditional notion that most neurons express a single classical neurotransmitter, the neurotransmitter co-release, often activity-dependent, has been increasingly recognized as an important mechanism fine-tuning the local synapses.^38^ Our finding of an increased ratio of glutamatergic to GABAergic signaling in SOM + interneurons adds to the growing evidence that neurotransmitter co-release underlies disorders of abnormal neural circuitry.^39^ This may also explain why anticonvulsants targeting GABAergic transmission are ineffective in epilepsy due to CD. Further, our findings of GABA/glutamate co-transmission in response to most but not all stimulations suggest that only a subset of SOM + interneurons gain the co-release capability, though the signature of neurotransmitter co-release warrants further investigations with tools such as paired recordings. Other possible mechanisms underlying the excitatory switch of interneurons include disinhibitory connections and depolarizing GABAergic currents,^28^ which was considered less likely given our data showing monosynaptic transmissions and an unaltered E_GABA_ in CD rats. Altogether, our findings expand the classic excitation/inhibition imbalance theory underlying epileptic seizures by highlighting the pro-convulsant role of SOM + interneurons (which are conventionally regarded as inhibitory), caused by neurotransmitter phenotype switching in late-stage CD.

In conclusion, our study illustrates the long-held observation of a widespread seizure network in late-stage CD (Supplementary Fig. S13). The results provide evidence that SOM + interneurons in the DG are crucial elements driving the large-scale seizure network. We also propose glutamate co-release as a novel mechanism underlying the SOM + interneuron deficits. Further evidence from the clinic, including advanced neuroimaging or surgical specimens, may be needed to verify the co-release in patients. Overall, our results identify hippocampal SOM + interneurons to be a potential therapeutic target in seizures due to cortical malformations.

## Materials and methods

### Ethics

For the human study, the institutional review board of the Second Affiliated Hospital School of Medicine Zhejiang University approved this study (license ID: 2020-235). A written patient consent was obtained from all included patients with SEEG implantation. All animal care regimens and experiments were approved by the Animal Care and Use Committee of Zhejiang Chinese Medical University, and were in complete compliance with the National Institutes of Health Guide for the Care and Use of Laboratory Animals.

### Patients

FCD cases were retrospectively recruited in the epilepsy center, the Second Affiliated Hospital of School of Medicine, Zhejiang University from January 2014 to June 2022. The inclusion criteria were as follows: all patients (1) underwent presurgical SEEG recordings; (2) underwent epilepsy surgery with pathologically diagnosed FCD I/II, according to the 2011 International League Against Epilepsy FCD classification.^40^ At the time of surgery, all patients had a comprehensive evaluation including a detailed clinical history, neurological examination, routine MRI, surface EEG and fluorodeoxyglucose-positron emission tomography. A decision to proceed to SEEG was made at the individual level when noninvasive data was discordant and typically when the presumed epileptogenic zone could not be confidently limited to a single lobe. The surgical plans were made with a multidisciplinary team. Patients were followed up for seizure relapses after surgery. Freedom from seizures corresponded to Engel classification I at the 1-year follow-up.^41^ Acute postoperative seizures during the first week were not taken into consideration.

### SEEG recording and analysis

We further selected patients who underwent SEEG exploration to quantify the seizure network in FCD subtypes. Intracerebral multiple contact electrodes (8–16 contacts, 2 mm in length, separated by 1.5 mm, and 0.8 mm in diameter, HKHS, Beijing, China) were placed according to the Talairach stereotactic method.^42^ The anatomical targeting of electrodes was established according to information from the noninvasive study and clinical hypotheses about the localization of the EZ. A postoperative CT scan or MRI was used to verify the position of electrodes. SEEG signal was recorded by a 256-channel long-term monitoring system (Nihon-Kohden EEG-1200C, Tokyo, Japan) with sampling rate at 2000Hz, and a SEEG bipolar montage was created and applied to all channels. Channels showing artifacts were excluded from analysis. Two electroclinical seizures with both video and SEEG data were analyzed for each patient. For patients with >2 captured seizures, all seizures were reviewed, of which 2 representative seizures were analyzed, so as to keep the same proportion of each distinct electroclinical seizure type. Seizure onset was manually defined as the first unequivocal SEEG signal change from the background in the setting of a sustained rhythmic discharge,^43^ and the first 15 s of SEEG onset were used for further analysis. Seizure onset without a fast discharge was excluded from subsequent analysis. We computed EI for all selected seizures using AnyWave Software (available at http://meg.univ-amu.fr/wiki/AnyWave). It is a normalized value that ranges from 0 (no epileptogenicity) to 1 (peak epileptogenicity). Electrodes with an EIå 0.3 is considered to have a high epileptogenicity.^11^ The number of brain regions/lobes with a high epileptogenicity is used to denote the extension of the seizure network.

### Animals

Timed pregnant (embryonic day 17–18, E17–18) female Sprague-Dawley rats of 8-9 weeks old were anesthetized with isoflurane (1.5 ml/min of oxygen and 3.5% isoflurane). We established the prenatal freeze lesion model as adapted from Takase et al.^20^ In brief, uterine horns were exposed. A liquid-nitrogen-cooled bronze probe was applied longitudinally on 2 points on the scalp of a rat embryo, from outside of the uterus wall. The uterus was then returned to the abdominal cavity. The freeze-lesioned pups were born at around E22 and weaned at postnatal day 21 (P21). Pups of time-pregnant rats not exposed to freeze lesioning were used as age-matched controls. All behavioral experiments were performed at around P50.^44^ No statistical method was used to predetermine sample size; sample sizes were estimated based on our previous studies for similar types of behavioral, biochemical, and electrophysiological analyses.^24,34^ No method of randomization was used.

### Electrode implantation, seizure induction and EEG recordings

Bipolar electrodes (each 0.2 mm in diameter; A.M. Systems, USA) were implanted into the right hippocampus (AP −5.28 mm, L −5.0 mm, V −6.0 mm) and one screw was placed over the motor cortex (AP: + 2.5 mm; ML: −2.5 mm) to record hippocampal and cortical EEGs respectively. Another screw was placed over the cerebellum to serve as the ground reference. All the above coordinates were measured from bregma according to the *Paxinos and Franklin’s Rat Brain Atlas*.^45^ Rats were allowed for recovery for 1 week after surgery.

To test the seizure severity, single-dose PTZ was injected intraperitoneally for seizure induction (60 mg/kg PTZ was used unless otherwise specified). Continuous EEG recording of each rat started 10 min before the PTZ injection and lasts for an additional 30 min using PowerLab system (AD Instruments, Australia).^34^ Ictal events were defined electrographically as a spike in frequency (≥2 Hz), high amplitude (>3 x baseline), rhythmic epileptiform, activity with a minimum duration of 10 s.^46^ Seizure severity was scored according to a modified Racine’s scale:^47^ 1, mouth and facial movement; 2, head nodding; 3, forelimb clonus. 4, rearing with forelimb clonus. 5, rearing and falling with forelimb clonus. 6, fully tonic-clonic generalized seizure or death. Stages 1–3 were focal seizures and stages 4–6 were considered GS. A trained observer who was unaware of the experimental groupings scored the seizure severity. All data were collected and analyzed in a double-blind manner.

### Viral delivery

The stereotactic viral delivery was strictly performed according to our previous studies.^48^ Briefly, anesthetized rats were mounted in a stereotaxic apparatus (Stoelting, USA). Virus was injected into the DG (AP: −3.0 mm, ML: −1.5 mm, V: −4.2 mm) with a 1-μL microliter syringes (Gaoge, China) controlled by an injection pump (Micro 4, World Precision, USA) at 100 nl/min. AAV-SOM-Cre and AAV-PV-Cre were purchased from BrainVTA Co., Ltd (Wuhan, China). All other viruses were purchased from OBiO Technolog Corp.,Ltd. (Shanghai, China).

For fluorometric monitoring the calcium activity of DG SOM + or PV + interneurons during seizures, a viral cocktail (1:1, 1 μl) of AAV-SOM-Cre (serotype: AAV2/9, viral titers: 2.50 × 10^12^ vg/mL) or AAV-PV-Cre (serotype: AAV2/9, viral titers: 3.38 × 10^12^ vg/mL) and AAV-EF1a-DIO-GCaMP6s (serotype: AAV2/9, viral titers: 2.05 × 10^12^ vg/mL) was stereotactically injected.^25^

To optogenetically inhibit SOM + or PV + interneurons in the DG, a viral cocktail of (1:1, 1 μl) of AAV-SOM-Cre or AAV-PV-Cre and AAV-CAG-FLEX-ArchT-GFP (serotype: AAV2/8, viral titers: 1.3 × 10^13^ vg/mL) was stereotactically injected.

To optogenetically activate SOM + or PV + interneurons in the DG, a viral cocktail of (1:1, 1 μl) of AAV-SOM-Cre or AAV-PV-Cre and AAV-EF1a-DIO-hChR2(H134R)-EYFP (serotype: AAV2/8, viral titers: 1.58 × 10^13^ vg/mL) was stereotactically injected.

### Fiber/cannula implantation

Stereotactic fiber/cannula implantation surgery on rats was described in detail in our previous study.^48^ For fluorometric monitoring, an optical fiber (300 μm O.D., 0.37 mm FOC-C-1.25-200-0.37-6.0, Inper, China) was implanted into the DG. For in vivo optogenetic manipulation, the guide cannulas (RWD, China), used to aid in optical fiber (diameter, 200 μm; Thorlabs, USA) insertion in optogenetic studies, were implanted in the DG. Electrode location and viral expression were histologically verified in all animals, and only rats with correct locations were taken into analysis. Co-labeling with anti-SOM or anti-PV antibodies was further performed in randomly selected subjects to verify the sensitivity and specificity of the viruses.

### Fiber photometry

Fiber photometry was performed as our previous study.^15^ Briefly, the fiber photometry system (Nanjing-Thinkertech, China) used a 488-nm diode laser (Coherent, USA), reflected by a dichroic mirror (Thorlabs, USA) and coupled into the optical fiber using a x10 objective lens (Olympus, USA) and fiber launch (Thorlabs, USA). The laser intensity at the interface between the fiber tip and the animal ranged from 0.01–0.03 mW to minimize bleaching. The GCaMP fluorescence was bandpass filtered (Thorlabs, USA) and collected by a photomultiplier tube (Hamamatsu, Japan). An amplifier (Hamamatsu, Japan) was used to convert the photomultiplier tube current output to voltage signals, which was further filtered through a low-pass filter (100 Hz cut-off). Photometry data were exported to MATLAB Mat files for further analysis. We segmented data based on individual trial of seizures and derived the values of fluorescence change (ΔF/F) by calculating (F − F0)/F0, which were presented with heatmaps or average plots. Only rats with GCaMP expression within the target region (DG) were taken into analysis (Supplementary Fig. S6).

### Photostimulation

Photostimulation was applied according to the previous report.^15^ Blue (473 nm) or yellow (589 nm) laser light was delivered through a 200 μm diameter optic fiber connected to the laser (BL473T3-050 or YL589T3-050, Shanghai Laser & Optics Century, China). The optic fiber was flat cut, and the laser power was adjusted to about 5 mW. The blue light (473 nm, 20 Hz, 10 ms/pulse) or DC yellow-light (589 nm) stimulation was delivered immediately after PTZ injection.

### Histology and quantification

The immunohistochemistry was performed strictly according to our previous studies.^48^ Briefly, rats that had undergone behavioral analysis were deeply anesthetized with pentobarbital (50 mg/kg, i.p.). We removed the brains and obtained coronal 20-μm sections with a sliding freezing microtome (Leica, Japan). Free-floating samples were used for Fig. 3f. Slide-mounted sections were used for the rest of figures. We processed sections for immunofluorescence for PV (1:400, Swant PV27), SOM (1:400, Santa cruz sc-13099), NeuN (1:400, Millipore MABN140), CaMKII (1:300, Invitrogen 13-7300) by incubating the sections with primary antibodies, and then incubated with a Alexafluor 488 conjugated secondary fluorescent antibody (1:400, Molecular Probes, USA) at room temperature. Fluoroshield Mounting Medium with DAPI (Sigma-Aldrich, USA) was used as a nuclear stain. We assessed the immunofluorescence with Leica SP8 laser confocal microscope.

Image analyses and quantification were performed using ImageJ (version 1.52a) software. For assessing the cell numbers, two coronal sections showing dorsal (Bregma −2.4 mm to −2.8 mm) and ventral hippocampus (Bregma −3.2 mm to −4.0 mm) were chosen. The data were averaged per rat. The number of rats used in each experiment was indicated in figures.

### Immunofluorescence staining for neuronal apoptosis

To better assess neuronal apoptosis, immunofluorescent double staining of TUNEL and SOM was conducted to determine the colocalization of apoptotic cells and SOM + neurons. In brief, frozen sections were immunostained with SOM (1:400, Santa cruz sc-13099) at 4 °C overnight and subsequently subjected to TUNEL staining using an In Situ Cell Death Detection kit (Roche, South San Francisco, CA, USA) according to the manufacturer’s suggested protocol. Finally, the sections were covered with 4′,6-diamidino-2-phenylindole (DAPI, Invitrogen). Positive cells were calculated per square millimeter from four random microscopic fields of each section (two sections per animal) under a fluorescence microscope (Olympus). The results were presented as the apoptotic ratio of the total neurons (SOM-TUNEL double stained cells/SOM-stained cells).

### Fluorescence in situ hybridization (FISH) by RNAscope

Rats were perfused with saline and 4% paraformaldehyde in PBS (pH 7.4). The harvested brains were fixed in 4% paraformaldehyde for another day before consecutive dehydration in 10, 20, and 30% sucrose. Fresh frozen rat brain slices with 14-μm thickness were subjected for FISH. RNAscope Multiplex Fluorescent Reagent Kit v2 (Advanced Cell Diagnostics, USA) was used for duplex hybridization by combing the CaMKII probe (ACDBio, 445231) with a VGAT probe (ACDBio, 319191). For further double labeling that combines FISH and immunofluorescence, the slices were then incubated with antibodies against SOM (1:400, Santa cruz sc-13099). We obtained images using Leica SP8 laser confocal microscope.

### In vitro electrophysiology

In vitro electrophysiology was strictly performed as previously reported.^24,49^ To obtain acute hippocampal slices, rats were anesthetized and decapitated, the brains were then quickly removed and submerged in oxygen-saturated artificial cerebrospinal fluid (ACSF) containing in mM: 120 NaCl, 11 Dextrose, 2.5 KCl, 1.28 MgSO_4_, 3.3 CaCl2, 1 NaH2PO_4_, and 14.3 NaHCO_3_. Coronal slices (300 μm) were cut using a vibratome (Leica, Germany) and incubated at 25°C for 1 h. The slices were transferred into a recording chamber at 25 °C for patch clamp recording. The glass patch pipettes (4–8 MΩ resistance) were pulled by a two-stage puller (PC-10, Narishige). For action potential recordings, the intracellular solution contained (in mM): 35K-gluconate, 110KCl, 104-(2-hydroxyethyl)-1-piperazineethanesulfonic acid (HEPES), 2MgCl_2_, and 2Na_2_ATP, 10ethylene glycol tetra-acetic acid (pH 7.4). For AP properties, step depolarized currents were injected through the pipettes in 5pA increments from 0pA to 100pA. We analyzed the first spike induced by the minimum depolarizing current (Rheobase) for threshold, amplitude, and the half-width. AP amplitude was defined as the voltage from the AP threshold to the AP peak, and the AP half-width was calculated as the duration at half-maximal amplitude.

For gramicidin-perforated patch-clamp recording, pipettes were back-filled with the same intracellular solution containing gramicidin (50 μg/ml) prepared freshly prior to recording. GABA_A_ receptor-mediated postsynaptic currents were isolated with AMPA receptor antagonist CNQX (20 μM) and NMDA receptor antagonist D-AP5 (50 μM). Whole-cell gramicidin perforated patch-clamp recordings were performed from visually and electrophysiologically identified granule cells in the DG. GABA_A_ receptor-mediated IPSCs were evoked through a concentric bipolar electrode placed 50–100 μm lateral to the recorded neuron (stimulation rate 0.1 Hz at 100 μA, and 100 μs duration). To identify the E_GABA_, the holding potential was systematically varied from −80 to 30 mV in 10-mV steps at the same time as the stimulation. The membrane potential was verified after breaking the perforated patch following the recording.

To record evoked synaptic currents, a low divalent ion ACSF (in mM: 125 NaCl, 3.5 KCl, 1.25 NaH_2_PO_4_, 0.5 MgCl_2_, 26 NaHCO_3_, 25 Dextrose, and 1 CaCl_2_) and cesium-based internal fluid (in mM: 100 CsCH_3_SO_3_, 20 KCl, 10 HEPES, 4 Mg-ATP, 0.3 Tris-GTP, 7 Tris_2_-Phosphocreatine, and 3 QX-314) were used. To examine whether the granule cells in the DG received excitatory synaptic input from SOM + interneurons, we injected a viral cocktail of (1:1, 1 μl) of AAV-SOM-Cre and AAV-EF1a-DIO-hChR2(H134R)-EYFP into the DG. Three weeks after viral injection, we performed whole-cell patch clamp recording. EPSCs were recorded at a holding potential of −60 mV and IPSCs were recorded at +10 mV.^24,49^ eEPSCs and eIPSCs were recorded from visually identified granule cells upon blue light stimulation (473 nm, 10 ms pulse width). Only granule cells with eIPSCs were considered having a direct connection with the stimulated SOM + interneurons, and were thereby included in the final analysis. To confirm whether the light-evoked currents were monosynaptic, TTX (1 μM) and 4-AP (100 μM) were applied. AMPA receptor antagonist CNQX (20 μM) and NMDA receptor antagonist D-AP5 (50 μM) were added to block excitatory synaptic transmission. All patch-clamp recordings were performed using an EPC10 patch-clamp amplifier (HEKA Instruments) with a low-pass filter at 3 kHz and a sample rate of 10 kHz.

### Statistical analysis

Statistical comparisons were performed using Graphpad Prism (version 9.0) with appropriate methods as indicated in the figure legends. Number of experimental replicates (n) is indicated in figures and refers to the number of experimental subjects used and independently treated in each experimental condition. For all experiments, at least two times experiment was repeated independently with similar results. Data were presented as number and percentages for categorical variables, and continuous data were expressed as mean ± s.e.m, unless otherwise specified. A two-tailed *P* value < 0.05 was considered statistically significant.

## Supplementary information

Supplementary materials

Data S1 source data

## Data Availability

The data that support the findings of this study are available as a supplementary spreadsheet of the article.
