# Supplementary material for: Excitatory somatostatin interneurons in the dentate gyrus drive a widespread seizure network in cortical dysplasia
Source: Signal Transduct Target Ther. 2023 May 17;8:186. doi: 10.1038/s41392-023-01404-9 (PMC10188524; doi:10.1038/s41392-023-01404-9)
Supplement: Supplementary file 1 — Supplementary materials [file 41392_2023_1404_MOESM1_ESM.docx]

Supplementary Materials for

**Excitatory Somatostatin Interneurons in the Dentate Gyrus Drive a Widespread Seizure Network in Cortical Dysplasia**

Yang Zheng, MD, PhD^1,2,3,4#^; Cenglin Xu, PhD^1,2,3,4#,^*; Jinyi Sun, MS^3^; Wenjie Ming, MD^4^; Sijie Dai, MS^3^; Yuying Shao, MS^3^; Xiaoyun Qiu, MS^2^; Menghan Li, MS^2^; Chunhong Shen, MD^4^; Jinghong Xu, MD^4^; Fan Fei, PhD^2,3^; Jiajia Fang, MD^5^; Xuhong Jiang, MD^1,2^; Guoqing Zheng, MD^1^; Weiwei Hu, PhD^3^; Yi Wang, PhD^1,2,3,4^; Shuang Wang, MD, PhD^4^; Meiping Ding, MD^4^*; Zhong Chen, PhD^1,2,3,4^*

Correspondence to: [chenzhong@zju.edu.cn](mailto:chenzhong@zju.edu.cn)

**This file includes:**

Figures. S1 to S13

Tables S1

**Other Supplementary Materials for this manuscript include the following:**

Data S1

Supplementary information accompanies the manuscript on the *Signal Transduction and Targeted Therapy* website http://www.nature.com/sigtrans

**
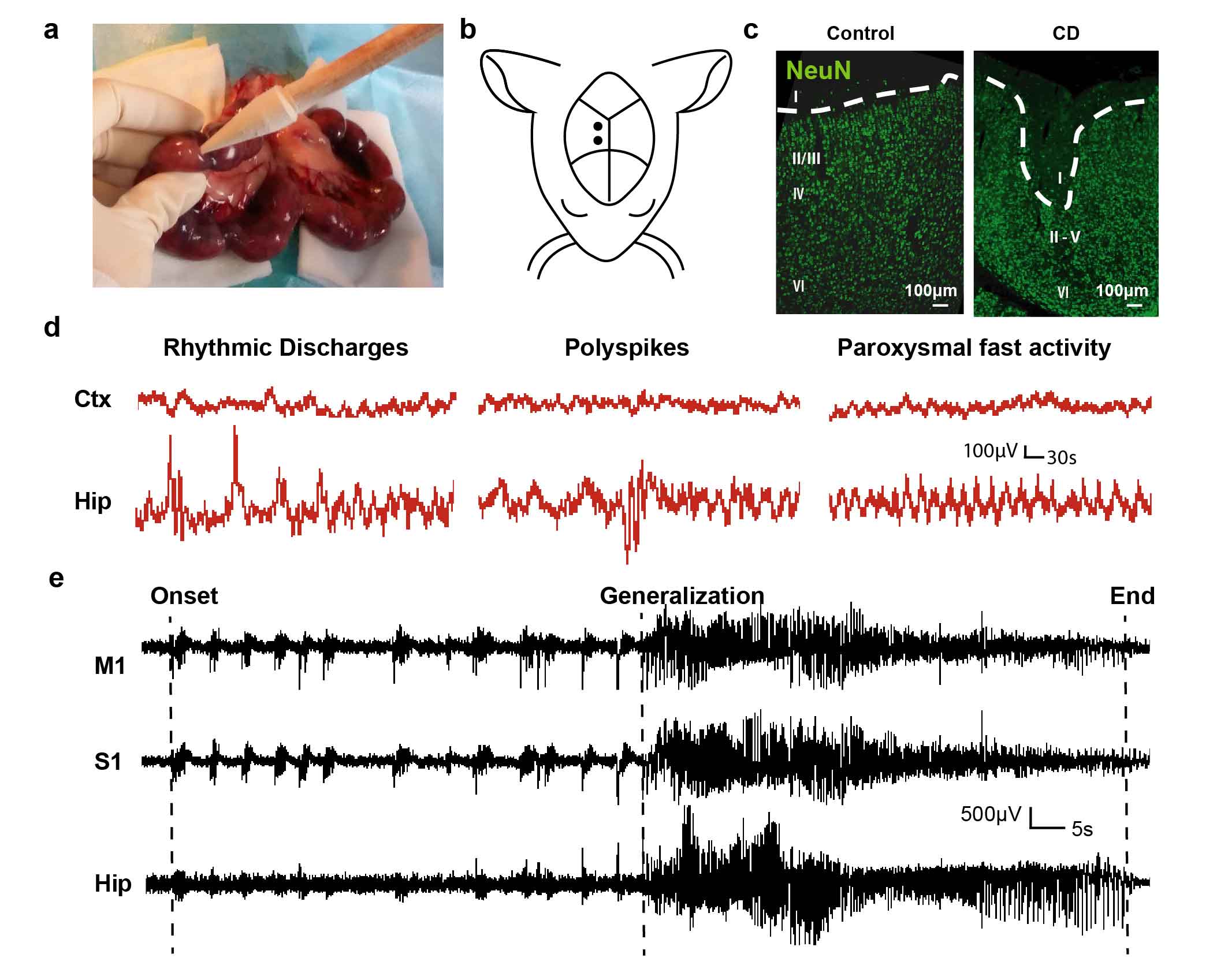
**

**Figure. S1.**

**The rat model of prenatal freeze lesioning.** a. Establishment of cortical dysplasia (CD) model in rats; b. Scheme of freeze lesioning; c. Microgyria lesions were detected in CD rats but not in controls; d. Spontaneous interictal epileptic discharges in CD rats, with a higher amplitude in the hippocampus (Hip) than the cortex (Ctx); e. Generalized ictal discharges could be induced with subthreshold pentylenetetrazol (PTZ).


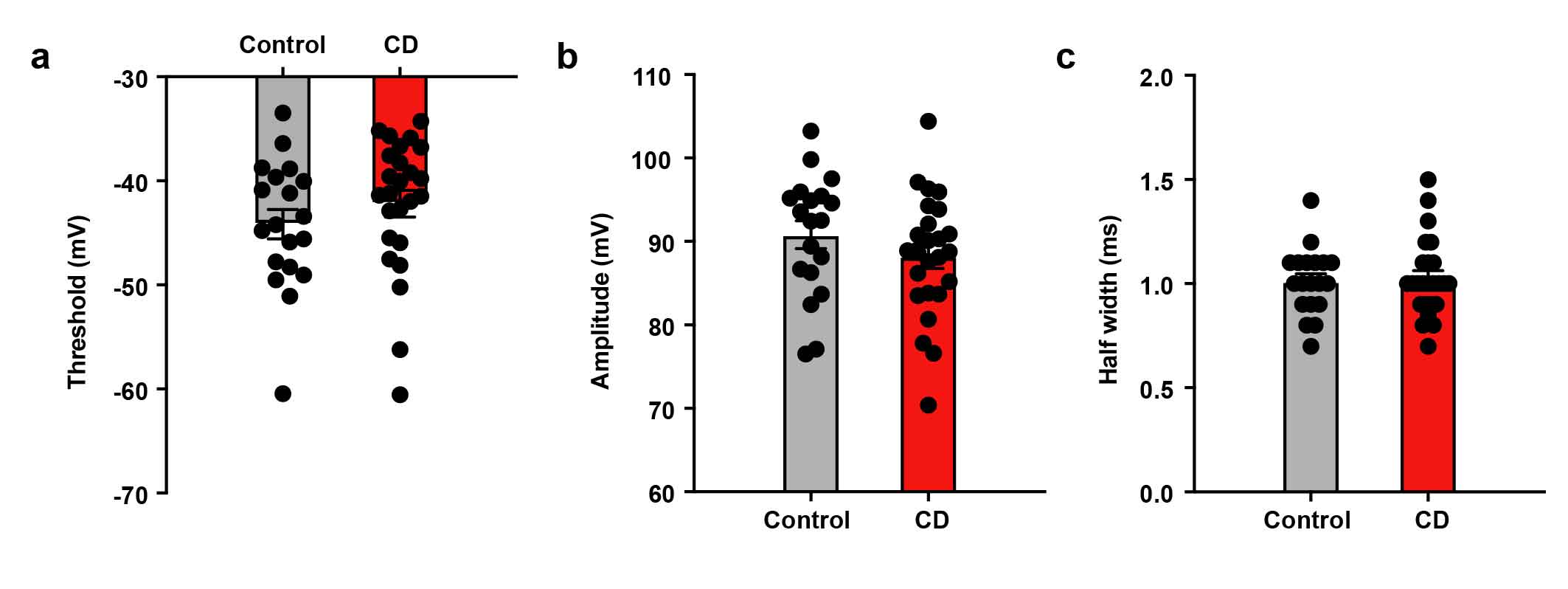


**Figure. S2.**

**Intrinsic membrane properties of hippocampal dentate gyrus granule cells.**

There was no significant differences in action potential threshold, amplitude and half-width in the granule cells between control and cortical dysplasia (CD) rats.

**
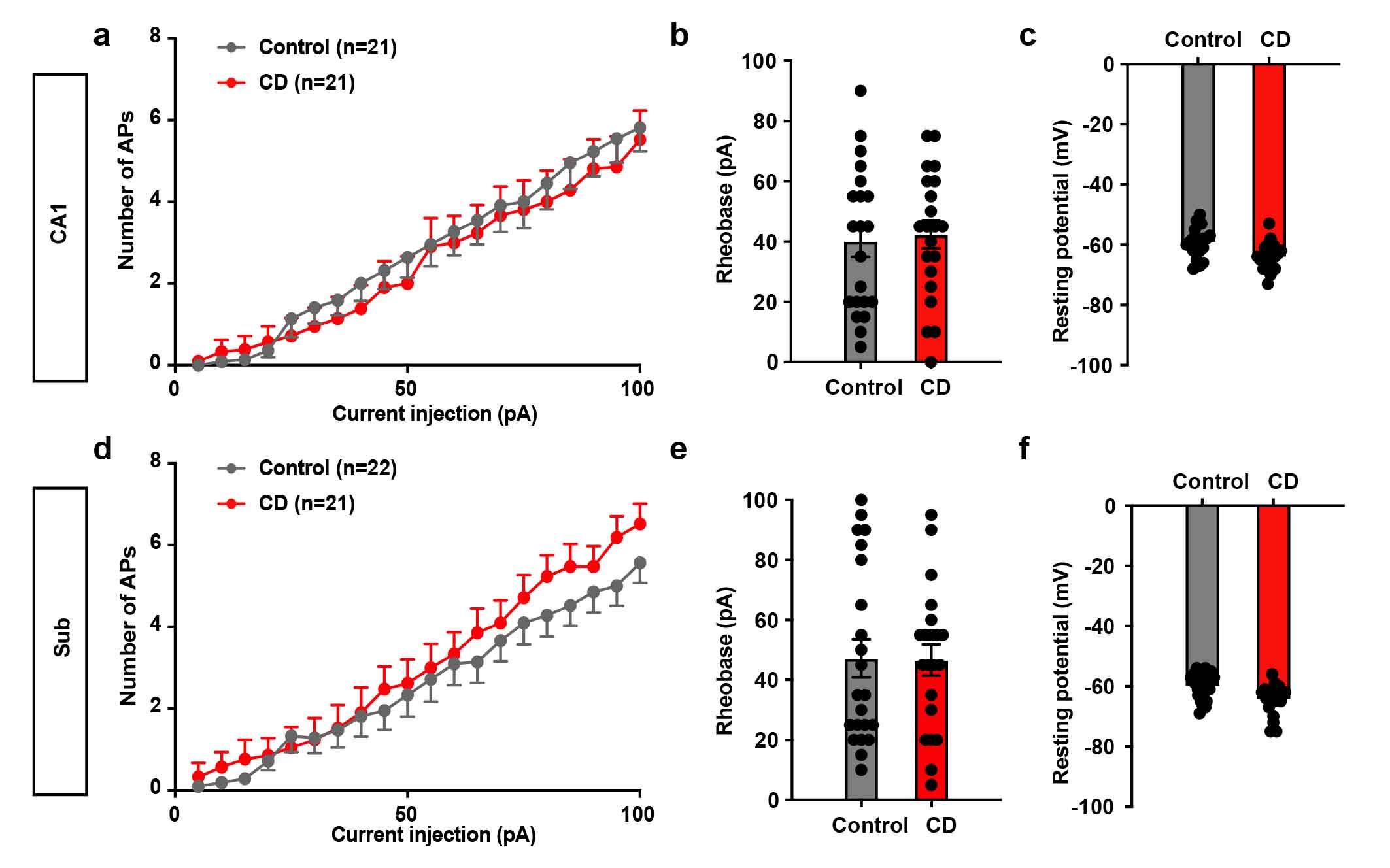
**

**Figure. S3.**

**Excitability of pyramidal cells in CA1 and subiculum of the hippocampus. (a, d)**. Similar number of spikes were evoked by trains of stepped depolarized currents in CA1 **(a)** and subiculum **(d)** pyramidal cells of cortical dysplasia (CD) and control rats; **(b, c, e, f**). The resting membrane potential and rheobase of CA1 **(b, c)** and subiculum **(e, f)** pyramidal cells were comparable between control and CD rats; Mann-Whitney U-test was used for **(b, c, e, f)**. Data are presented as mean ± s.e.m. and error bars represent s.e.m.

**
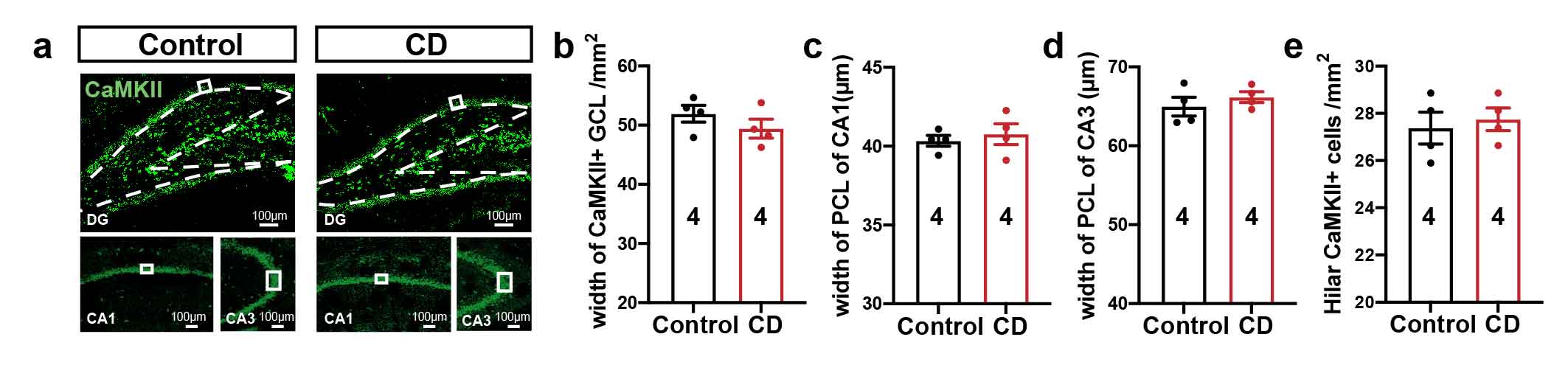
**

**Figure. S4.**

**Number of calmodulin-dependent protein kinase II (CaMKII)+ glutamatergic neurons in the hippocampus of control and cortical dysplasia (CD) rats.** a. Glutamatergic neurons within the hippocampus (dentate gyrus [DG], upper; CA1, lower left; CA3, lower right) were shown via CaMKII labeling (green); b-e. The width of CaMKII+ granule cell layer (GCL) **(b)**, pyramidal cell layer **(**PCL) of CA1 **(c)** and CA3 **(d)** and density of hilar CaMKII+ neurons **(e)** were comparable between control and CD rats. Mann-Whitney U-test was used. Data are presented as mean ± s.e.m. and error bars represent s.e.m.

**
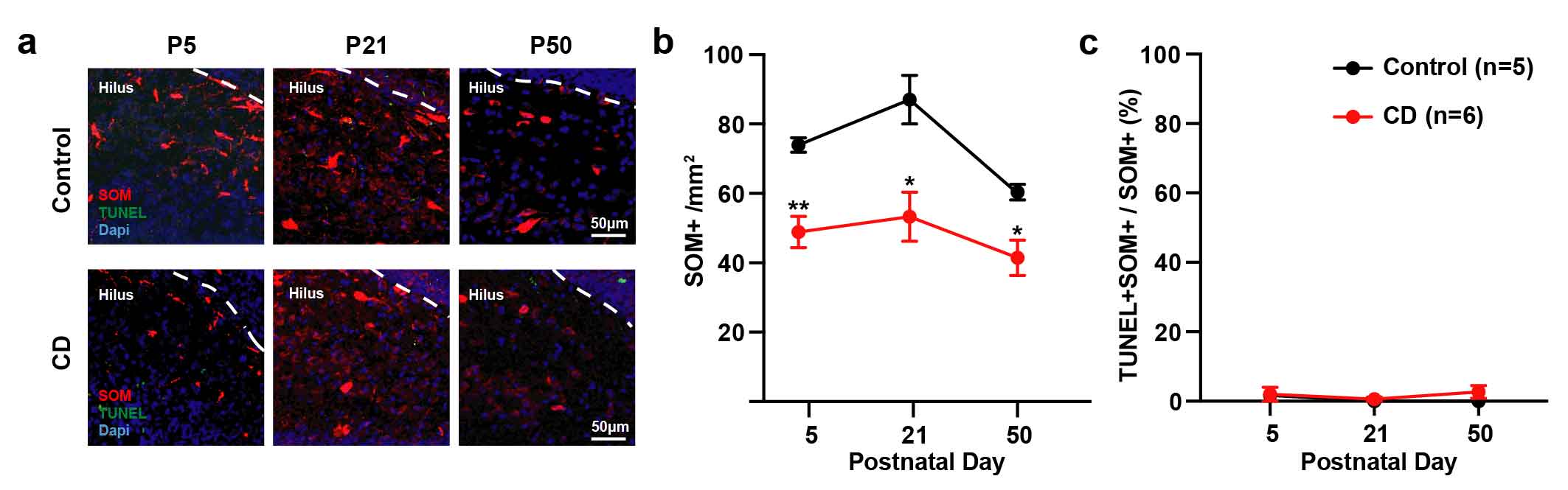
**

**Figure. S5.**

**Loss of somatostatin (SOM)+ interneurons and SOM apoptosis at different developmental stages.** a. Representative images of SOM-TUNNEL co-staining in the hilus of rats at different developmental stages; b. SOM+ interneurons in the DG were significantly reduced at postnatal day 5 (P5, at birth), P21 (when neuronal morphology and electrophysiological characteristics reach a plateau) and P50 (adulthood). **P* < 0.05, ** *P* < 0.01; c. There were scarce SOM+ interneurons co-expressing TUNEL at different developmental stages. Two-way ANOVA was used for **(b-c)**. Data are presented as mean ± s.e.m. and error bars represent s.e.m.

**
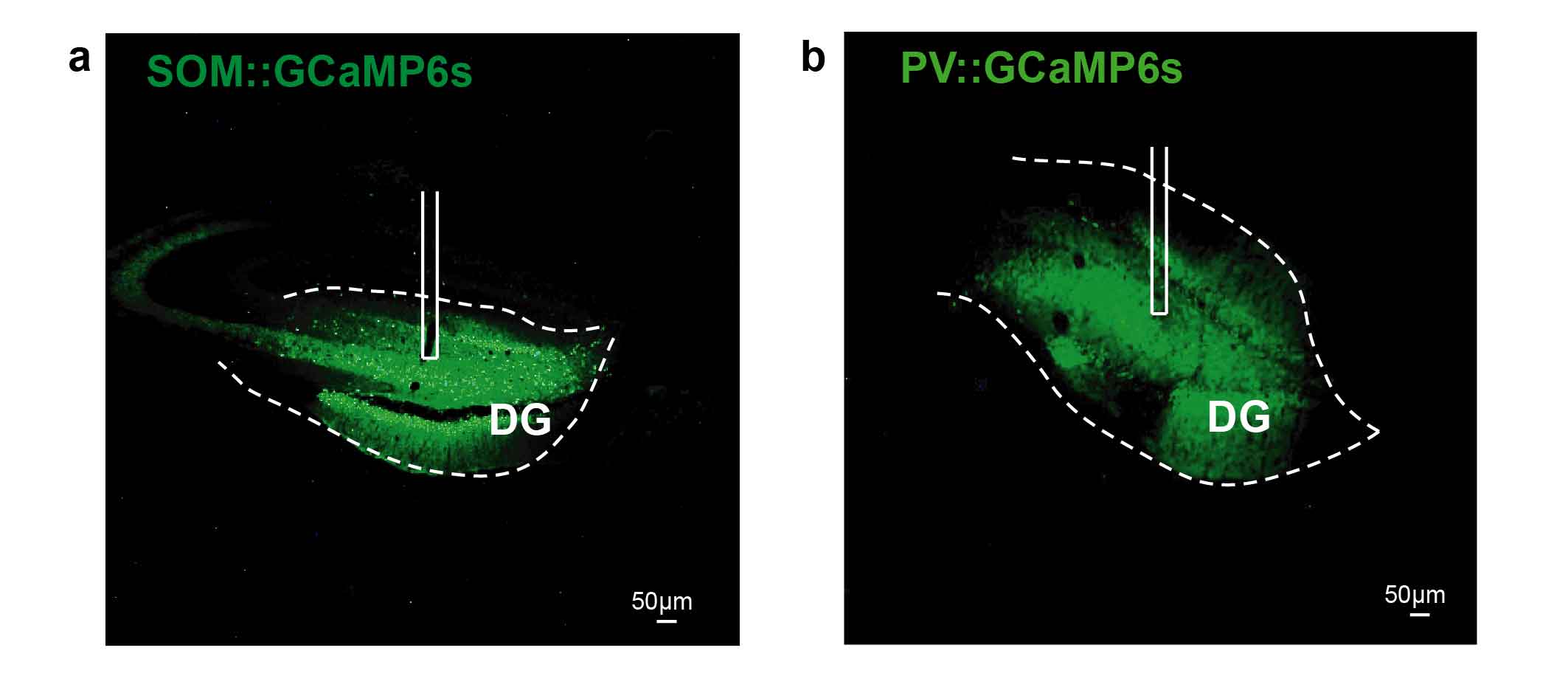
**

**Figure. S6.**

**Representative images of SOM::GCaMP6s and PV::GCaMP6s expression in fiber photometry.** Only rats with SOM::GCaMP6s **(a)** and PV::GCaMP6s **(b)** expression within the dentate gyrus (DG) were included in the final analysis.

**
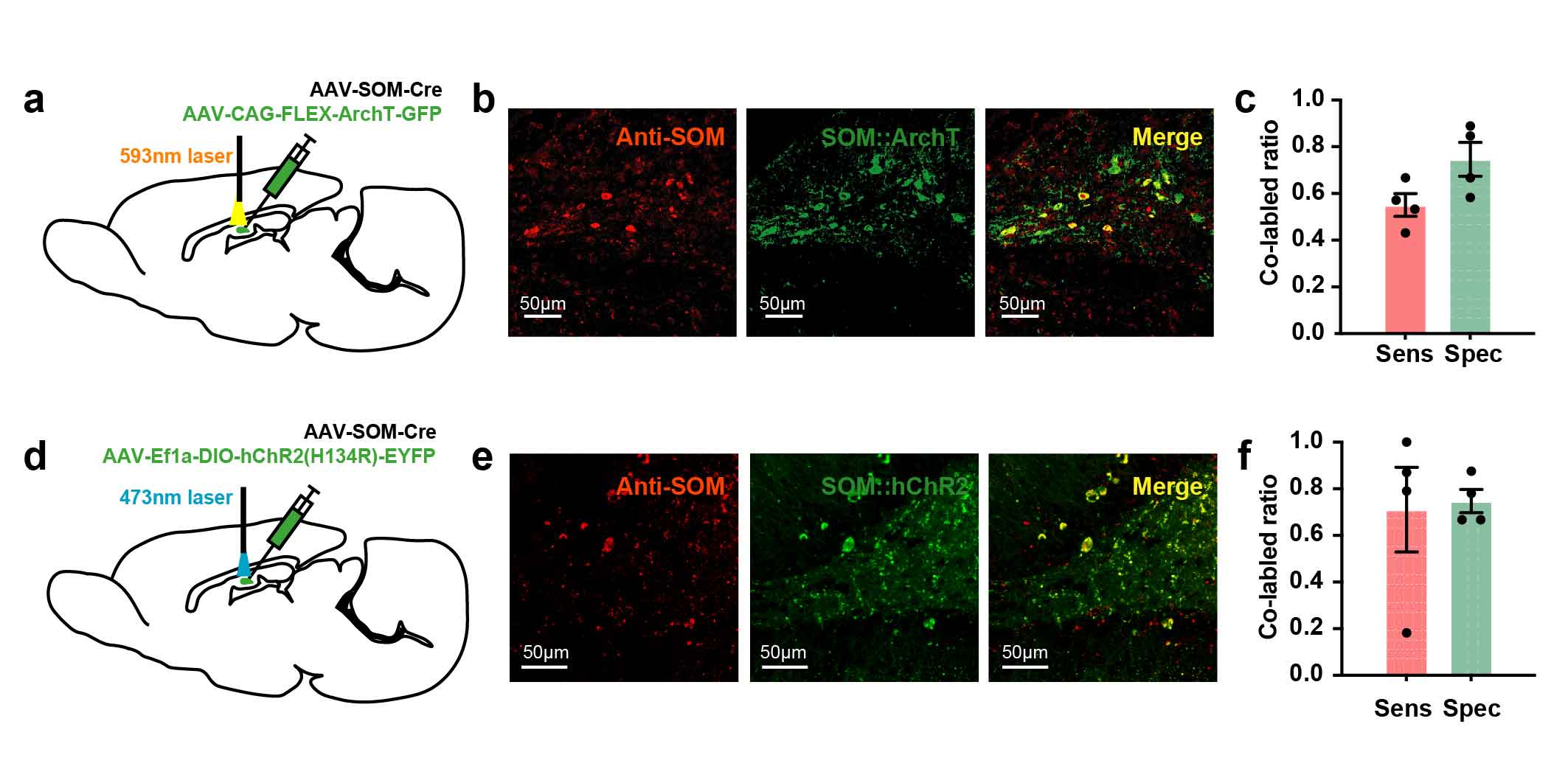
Figure. S7.**

**Verification of the sensitivity and specificity of AAV-SOM-Cre labeling. a** Scheme of experiment for viral cocktail (AAV-SOM-Cre and AAV-CAG-FLEX-ArchT-GFP) injection into the dentate gyrus (DG) for optogenetic inhibition studies. **b** Representative images of dentate gyrus showing the overlap (yellow) of SOM::ArchT (green) and somatostatin (SOM)+ neurons (red). **c** 55.07±4.87% of SOM+ neurons expressed ArchT, and 74.63±7.26% of ArchT+ neurons expressed SOM. **d** Scheme of experiment for viral cocktail (AAV-SOM-Cre and AAV-EF1a-DIO-hChR2(H134R)-EYFP) injection into the DG for optogenetic activation studies. **e** Representative images of DG showing the overlap (yellow) of SOM::hChR2 (green) and SOM+ neurons (red). **f** 71.05±18.14% of SOM+ neurons expressed hChR2, and 74.71±5.03% of hChR2+ neurons expressed SOM. Mann-Whitney U-test was used. Data are presented as mean ± s.e.m. and error bars represent s.e.m.

**
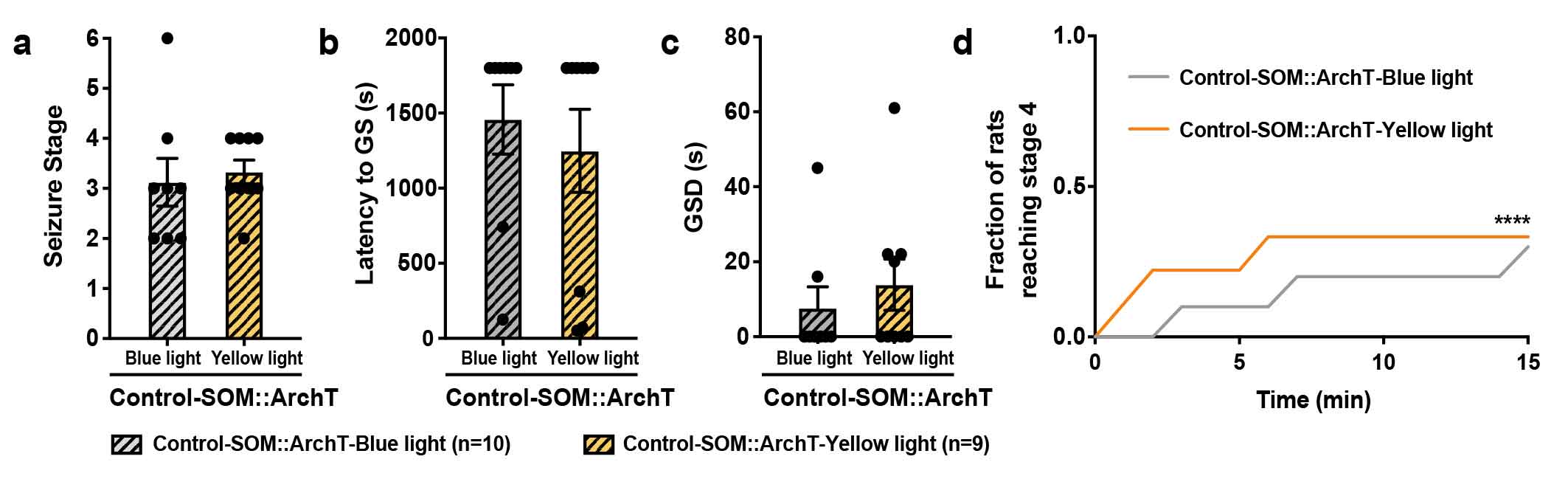
**

**Figure. S8.**

**Optogenetic inhibition of somatostatin (SOM)+ interneurons in control rats. a-c.** Optogenetic inhibition of dentate gyrus (DG) SOM+ interneurons in control rats failed to alter the seizure stage **(a)**, latency to generalized seizures (GS) **(b)**, and generalized seizure duration (GSD) **(c)**. **d.** Optogenetic inhibition of DG SOM+ interneurons in control rats significantly accelerated seizure progression. **** *P* < 0.0001, Mann-Whitney U-test was used for **(a-c)**. Two-way ANOVA was used for **(d)**. Data are presented as mean ± s.e.m. and error bars represent s.e.m.

**
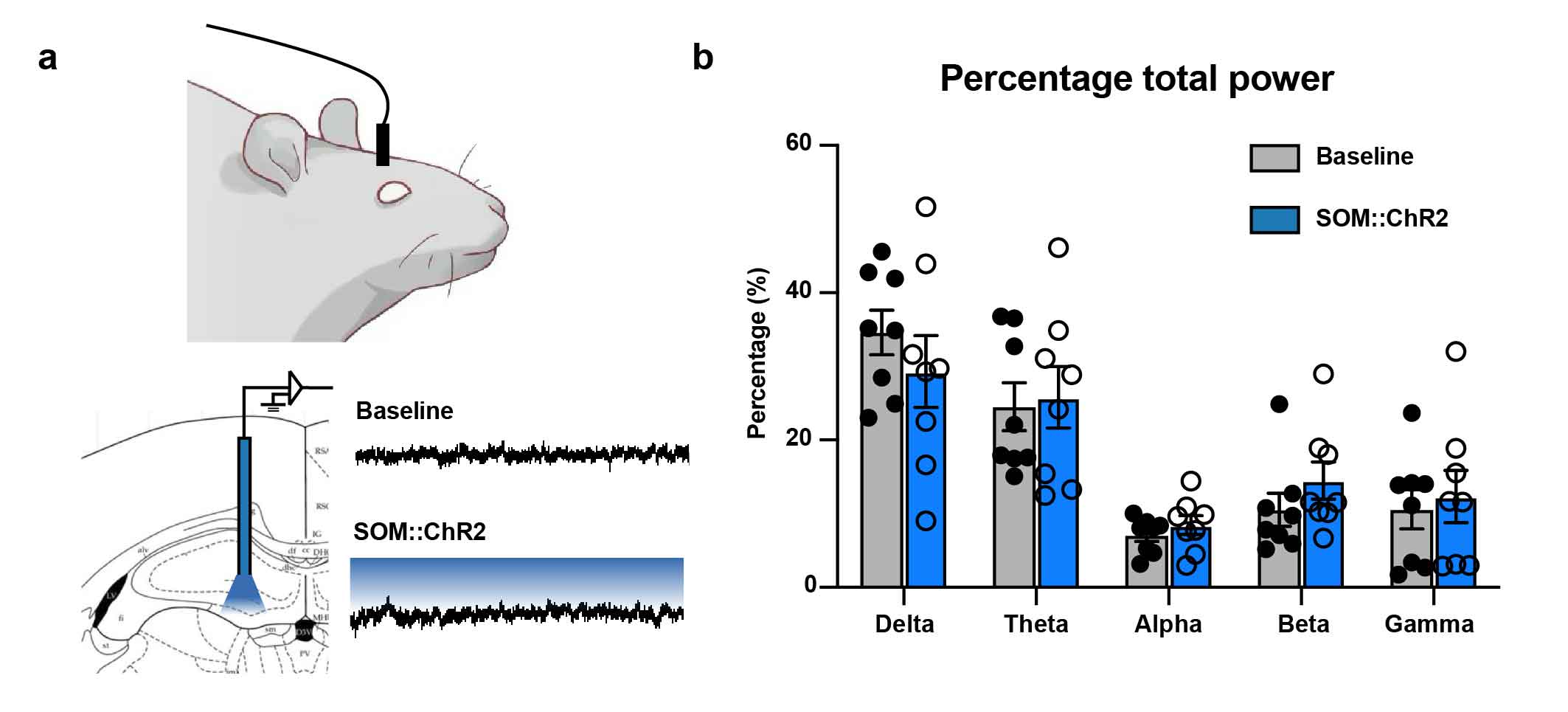
**

**Figure. S9.**

**Effects of somatostatin (SOM)+ activation on seizure initiation.** a. Experimental setup. The 10 seconds-epoch before and after blue light stimulation were analyzed. b. Spectral analysis of the percentage total power across the frequency bands before and after optogenetic activation of SOM+ interneurons. The frequency bands are represented as Delta (0-4Hz), Theta (4-8Hz), Alpha (8-12Hz), Beta (12-30Hz) and Gamma (30-100Hz). Mann-Whitney U-test was used. Data are presented as mean ± s.e.m. and error bars represent s.e.m.

**
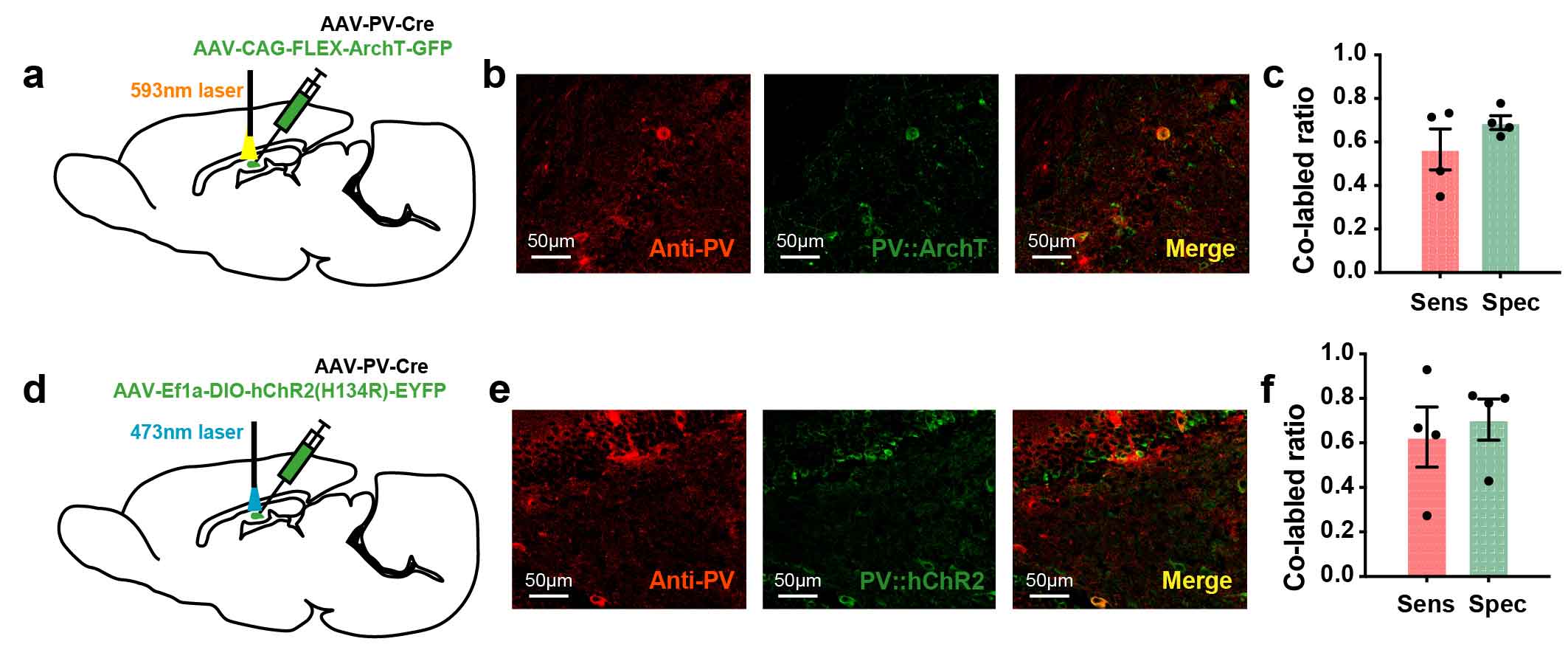
Figure. S10.**

**Verification of the sensitivity and specificity of AAV-PV-Cre labeling. a** Scheme of experiment for viral cocktail (AAV-PV-Cre and AAV-CAG-FLEX-ArchT-GFP) injection into the dentate gyrus (DG) for optogenetic inhibition studies. **b** Representative images of dentate gyrus showing the overlap (yellow) of PV::ArchT (green) and PV+ neurons (red). **c** 56.61±9.42% of PV+ neurons expressed ArchT, and 68.92±3.23% of ArchT+ neurons expressed PV. **d** Scheme of experiment for viral cocktail (AAV-PV-Cre and AAV-EF1a-DIO-hChR2(H134R)-EYFP) injection into the DG for optogenetic activation studies. **e** Representative images of DG showing the overlap (yellow) of PV::hChR2 (green) and PV+ neurons (red). **f** 62.61±13.48% of PV+ neurons expressed hChR2, and 70.47±9.23% of hChR2+ neurons expressed PV. Mann-Whitney U-test was used. Data are presented as mean ± s.e.m. and error bars represent s.e.m.

**
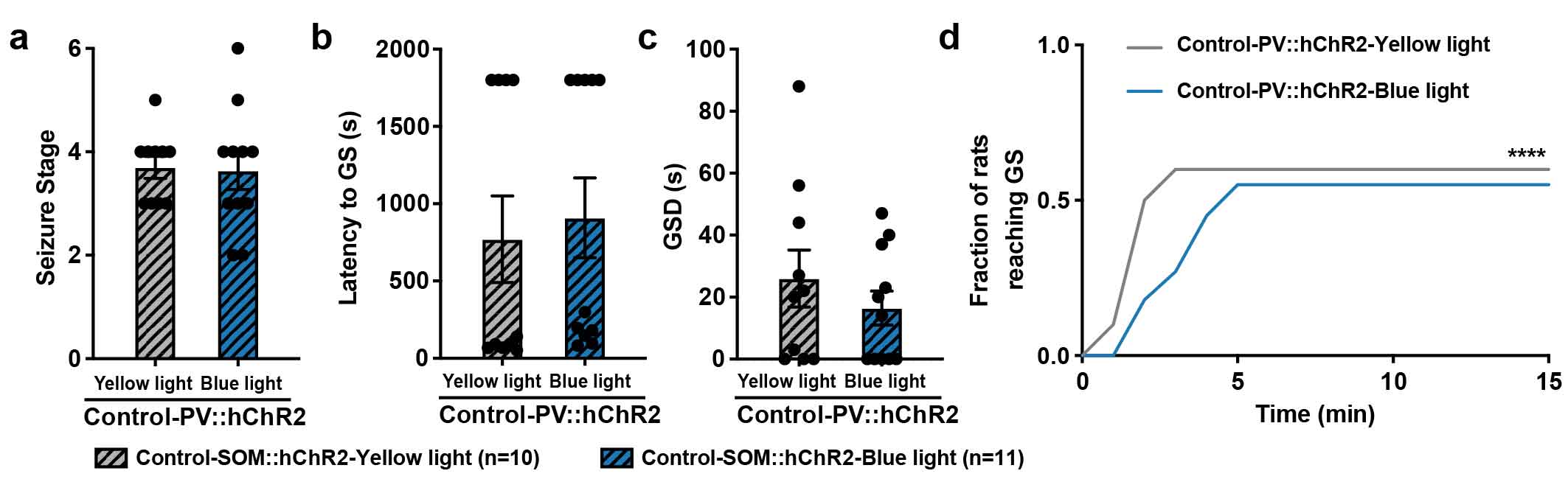
**

**Figure. S11.**

**Optogenetic activation of parvalbumin (PV)+ interneurons in control rats. a-c.** Optogenetic activation of dentate gyrus (DG) PV+ interneurons in control rats failed to alter the seizure stage **(a)**, latency to generalized seizures (GS) **(b)**, and generalized seizure duration (GSD) **(c)**. **d.** Optogenetic activation of DG PV+ interneurons in control rats significantly attenuated seizure progression. **** *P* < 0.0001, Mann-Whitney U-test was used for **(a-c)**. Two-way ANOVA was used for **(d)**. Data are presented as mean ± s.e.m. and error bars represent s.e.m.

**
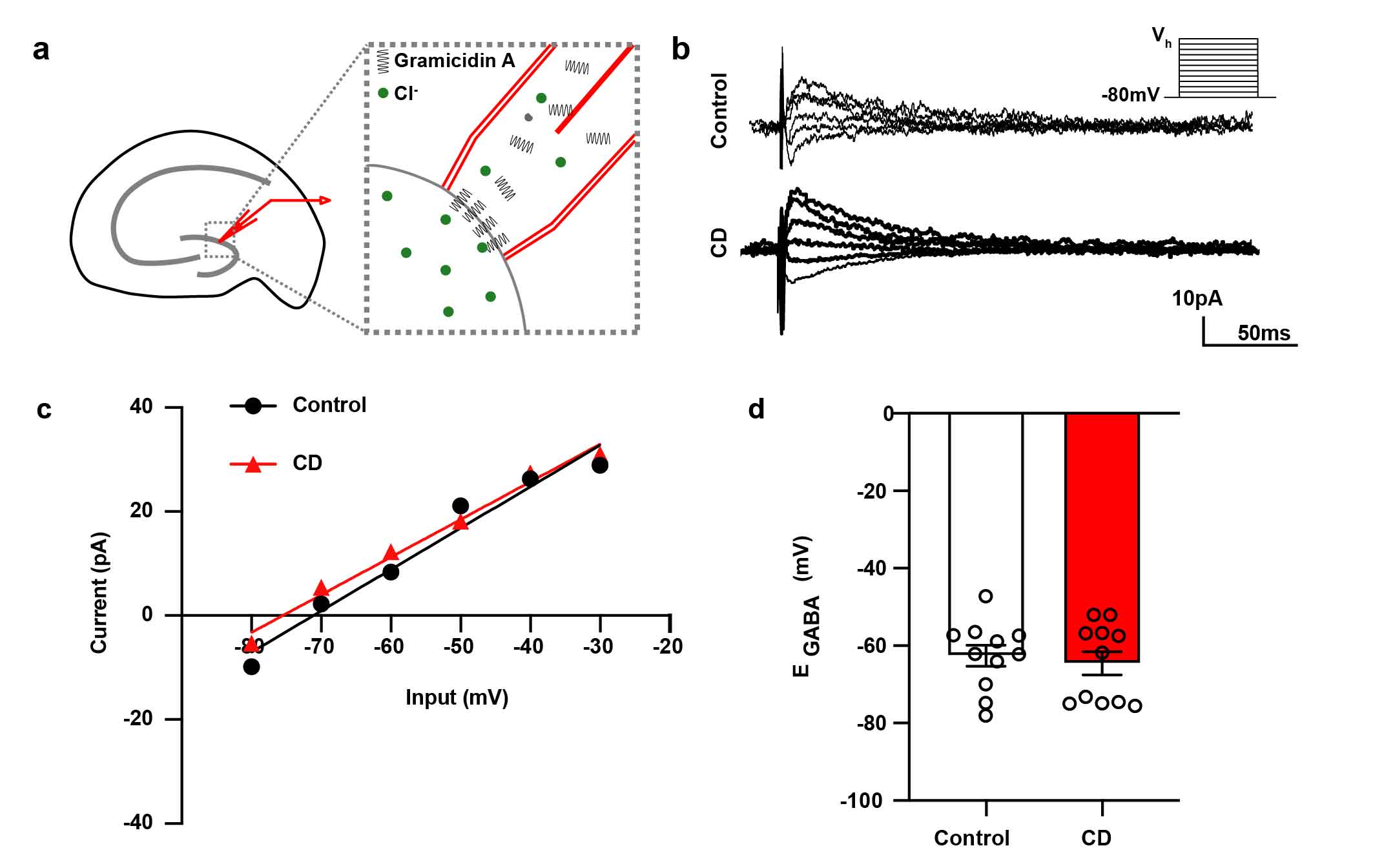
**

**Figure. S12.**

**Perforated patch recording of the equilibrium potential for GABA_A_ receptors (E_GABA_).** a. Scheme of gramicidin-perforated patch recordings in the granule cells of dentate gyrus (DG); b. Representative traces of reversal potential for GABAergic transmission in granule cells of DG in control and cortical dysplasia (CD) rats; c. Representative current-input relationship showing similar E_GABA_ between control and CD rats; d. E_GABA_ was comparable between control and CD rats. Mann-Whitney U-test was used. Data are presented as mean ± s.e.m. and error bars represent s.e.m.


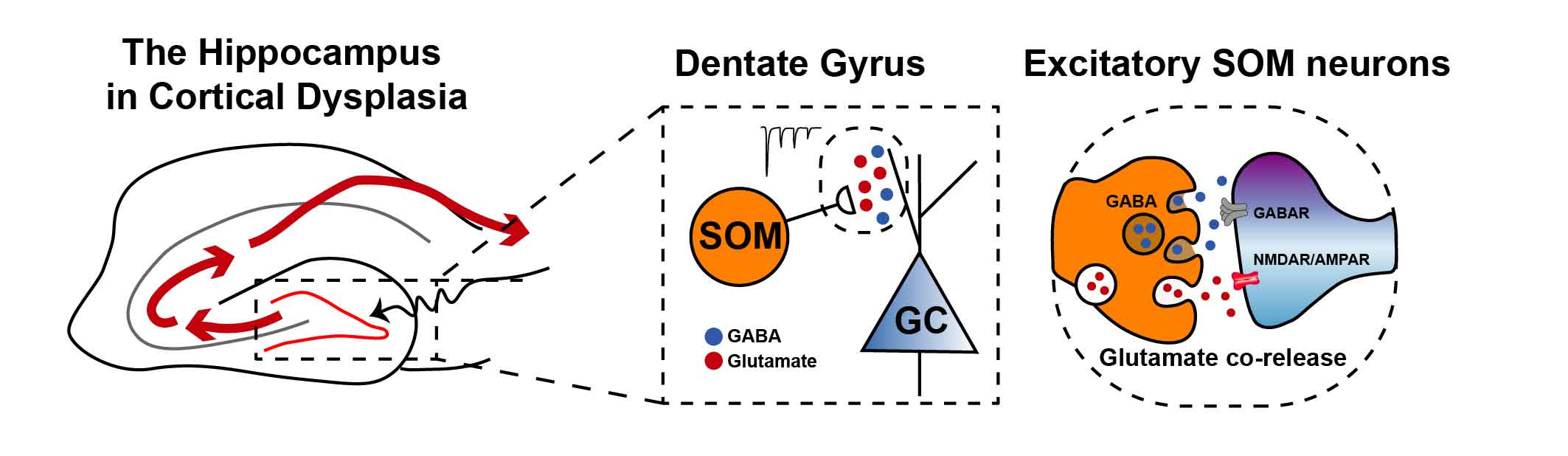


**Figure. S13.**

**Scheme of the neurotransmitter phenotype switch of somatostatin-positive interneurons in cortical dysplasia**

The hippocampus serves as an important brain region in the widespread seizure network of late-stage cortical dysplasia. Importantly, the excitatory somatostatin (SOM)+ interneurons in the dentate gyrus co-release glutamate, which turns on the excitatory switch of the hippocampus in cortical dysplasia.

**Supplementary Table S1. Characteristics of Patients with Cortical Dysplasia in the Stereo-electroencephalography Analysis**

| N | Sex | Age at SEEG, y | Pathology | SOP in Hippocampus | EI_hip_ | Type of surgery | Epilepsy onset, age, y | Duration, y | Engel class | Follow-up, mo |
| --- | --- | --- | --- | --- | --- | --- | --- | --- | --- | --- |
| 1 | F | 7 | FCD I | FA | 0.13 | Left occipital L | 4 | 3 | I | 16 |
| 2 | M | 26 | FCD I | LVFA | 0.7 | Right frontal L | 6 | 20 | I | 12 |
| 3 | M | 14 | FCD I | LVFA | 0.29 | Right insular L | 5 | 9 | I | 14 |
| 4 | M | 8 | FCD I | Beta sharp activities | 0.36 | Left amygdalohippocampectomy | 4 | 4 | I | 12 |
| 5 | M | 20 | FCD I | Rhythmic spikes or spike-waves, at low frequency and with high amplitude | 0.08 | Left amygdalohippocampectomy | 1 | 19 | II | 11 |
| 6 | M | 13 | FCD I | Preictal spiking with rhythmic spikes of low frequency followed by LVFA | 0.8 | Left frontal L | 8 | 5 | I | 25 |
| 7 | M | 33 | FCD I | Burst of polyspikes of high frequency and amplitude followed by LVFA | 0.78 | Right frontal L | 5 | 28 | I | 33 |
| 8 | F | 38 | FCD I | Rhythmic spikes or spike-waves, at low frequency and with high amplitude | 0.86 | Left amygdalohippocampectomy | 28 | 10 | II | 22 |
| 9 | M | 26 | FCD I | Slow wave or baseline shift followed by LVFA | 0.66 | Right amygdalohippocampectomy | 16 | 10 | IV | 18 |
| 10 | F | 20 | FCD I | LVFA | 0.05 | Left temporaloccipital L | 13 | 7 | II | 45 |
| 11 | F | 20 | FCD I | LVFA | 0.54 | Right anterior temporal lobe lobectomy | 3 | 17 | III | 50 |
| 12 | M | 20 | FCD I | Slow wave or baseline shift followed by LVFA | NA | Left frontal L | 8 | 12 | I | 31 |
| 13 | M | 12 | FCD I | Burst of polyspikes of high frequency and amplitude followed by LVFA | NA | Right parietal L | 21 | 9 | I | 57 |
| 14 | F | 27 | FCD I | Rhythmic spikes or spike-waves, at low frequency and with high amplitude | 0.34 | Right temporal L | 15 | 12 | NA | 10 |
| 15 | M | 8 | FCD I | delta brush | 0.03 | Right temporo-insular L | 6 | 2 | I | 12 |
| 16 | M | 21 | FCD I | Slow wave or baseline shift followed by LVFA. | NA | Right frontal L | 5 | 16 | NA | 9 |
| 17 | F | 26 | FCD I | LVFA | 0.1 | Right frontal L | 6 | 20 | I | 12 |
| 18 | M | 26 | FCD II | LVFA | 1 | Right temporal L | 21 | 5 | I | 17 |
| 19 | M | 9 | FCD II | FA | 0.18 | Right frontal L | 7 | 2 | I | 17 |
| 20 | M | 24 | FCD II | LVFA | NA | Right frontal L | 5 | 21 | III | 13 |
| 21 | M | 37 | FCD II | Preictal spiking with rhythmic spikes of low frequency followed by LVFA | NA | Right parietal L | 10 | 27 | I | 13 |
| 22 | M | 34 | FCD II | Preictal spiking with rhythmic spikes of low frequency followed by LVFA | 0.14 | Right frontal L | 11 | 23 | I | 24 |
| 23 | F | 17 | FCD II | LVFA | 0.72 | Right temporal L | 7 | 10 | I | 22 |
| 24 | M | 10 | FCD II | Burst of polyspikes of high frequency and amplitude followed by LVFA | NA | Left frontal L | 2 | 8 | I | 37 |
| 25 | M | 13 | FCD II | LVFA | NA | Right frontal L | 3 | 10 | I | 37 |
| 26 | M | 30 | FCD II | Burst of polyspikes of high frequency and amplitude followed by LVFA | NA | Right frontal L | 10 | 20 | I | 32 |
| 27 | F | 4 | FCD II | LVFA | NA | Right frontal L | 3 | 1 | III | 34 |
| 28 | F | 16 | FCD II | Burst of polyspikes of high frequency and amplitude followed by LVFA | NA | Left frontal L | 11 | 5 | I | 34 |
| 29 | M | 34 | FCD II | Rhythmic spikes or spike-waves, at low frequency and with high amplitude | 1 | Left parietal L | 14 | 20 | I | 31 |
| 30 | M | 29 | FCD II | Preictal spiking with rhythmic spikes of low frequency followed by LVFA | 0.5 | Right temporo-occipital L | 17 | 12 | I | 31 |
| 31 | F | 34 | FCD II | Preictal spiking with rhythmic spikes of low frequency followed by LVFA | 0.005 | Left temporo-occipital L | 12 | 22 | I | 42 |
| 32 | F | 4 | FCD II | Slow wave or baseline shift followed by LVFA | NA | Right frontal L | 1 | 3 | I | 46 |
| 33 | M | 6 | FCD II | LVFA | NA | Left temporo-parietal- occipital L | 3 | 3 | I | 42 |
| 34 | F | 23 | FCD II | LVFA | NA | Right parietal L | 7 | 16 | I | 56 |
| 35 | M | 26 | FCD II | Preictal spiking with rhythmic spikes of low frequency followed by LVFA | NA | Right frontal L | 24 | 2 | NA | 4 |
| 36 | M | 23 | FCD II | Preictal spiking with rhythmic spikes of low frequency followed by LVFA | NA | Left parieto-insular L | 3 | 20 | NA | 5 |
| 37 | M | 33 | FCD II | Burst of polyspikes of high frequency and amplitude followed by LVFA | NA | Right parietal L | 27 | 6 | NA | 5 |
| 38 | M | 23 | FCD II | Preictal spiking with rhythmic spikes of low frequency followed by LVFA | NA | Right frontal L | 14 | 9 | II | 12 |

Abbreviations: **FCD,** focal cortical dysplasia; **NA**, not applicable; **SEEG,** Stereo-electroencephalography ; **SOP,** seizure onset pattern; **FA,** fast activity; **LVFA,** low-voltage fast activity; **EI,** epileptogenicity index; **L,** lesionectomy

Data S1. (separate file)

Source data of all figures and supplementary figures.
